# Schwann cell reprogramming and lung cancer progression: a meta-analysis of transcriptome data

**DOI:** 10.18632/oncotarget.27204

**Published:** 2019-12-31

**Authors:** Victor Menezes Silva, Jessica Alves Gomes, Liliane Patrícia Gonçalves Tenório, Genilda Castro de Omena Neta, Karen da Costa Paixão, Ana Kelly Fernandes Duarte, Gabriel Cerqueira Braz da Silva, Ricardo Jansen Santos Ferreira, Bruna Del Vechio Koike, Carolinne de Sales Marques, Rafael Danyllo da Silva Miguel, Aline Cavalcanti de Queiroz, Luciana Xavier Pereira, Carlos Alberto de Carvalho Fraga

**Affiliations:** ^1^ Department of Medicine, Federal University of Alagoas, Campus Arapiraca, Brazil; ^2^ Department of Medicine, Federal University of the São Francisco Valley, Petrolina, Brazil

**Keywords:** bioinformatic, lung squamous cell carcinoma, lung adenocarcinoma, neuroactive ligand-receptor interaction

## Abstract

Schwann cells were identified in the tumor surrounding area prior to initiate the invasion process underlying connective tissue. These cells promote cancer invasion through direct contact, while paracrine signaling and matrix remodeling are not sufficient to proceed. Considering the intertwined structure of signaling, regulatory, and metabolic processes within a cell, we employed a genome-scale biomolecular network. Accordingly, a meta-analysis of Schwann cells associated transcriptomic datasets was performed, and the core information on differentially expressed genes (DEGs) was obtained by statistical analyses. Gene set over-representation analyses was performed on core DEGs to identify significantly functional and pathway enrichment analysis between Schwann cells and, lung adenocarcinoma (LUAD) and lung squamous cell carcinoma (LUSC). DEGs were further integrated with genome-scale human biomolecular networks. miRNAs were proposed by the reconstruction of a transcriptional and post-transcriptional regulatory network. Moreover, microarray-based transcriptome profiling was performed, and the prognostic power of selected dedifferentiated Schwann cell biomolecules was predicted. We observed that pathways associated with Schwann cells dedifferentiation was overexpressed in lung cancer samples. However, genes associated with Schwann cells migration inhibition system were downregulated. Besides, miRNA targeting those pathways were also deregulated. In this study, we report valuable data for further experimental and clinical analysis, because the proposed biomolecules have significant potential as systems biomarkers for screening or for therapeutic purposes in perineural invasion of lung cancer.

## INTRODUCTION

Peripheral nervous system plays an important role in neoplastic invasion. The glial cells present in this system, specifically Schwann cells, have been associated with the process known as perineural invasion. Perineural invasion occurs mainly in pancreas, prostate, breast, lung and head and neck cancers; and causes morbidity of patient through pain and paralysis, associated with a high risk of local recurrence and decreasing survival patients rates [1–3]. Understanding how cancer invades nerves is an essential step in developing more effective treatment strategies. The main questions are how the neoplastic cells interact with the cells of the nervous system and how they acquire mobile and invasive characteristics from these interactions.

Schwann cells performs several roles, depends on their ability to reversibly dedifferentiate and re-differentiate into subtypes with different phenotypes. After the nerve damage, Schwann cells become dedifferentiated, lose their capacity to myelinate and, promote neuronal orientation during repair [3]. This is accompanied by the reexpression of proteins lost during the myelinizing differentiation program, such as the glial fibrillary acidic protein (GFAP) and the neural cell adhesion molecule 1 (NCAM1) [1, 4]. Paracrine signaling has been implicated in perineural invasion, with factors secreted by nerves, including glial cell line derived neurotrophic factor (GDNF), increasing the invasion of tumor cells along the nerves [5, 6].

Schwann cells were identified in the tumor surrounding area prior to initiate the invasion process underlying connective tissue. These cells promote cancer invasion through direct contact, while paracrine signaling and matrix remodeling are not sufficient to proceed [4]. These cells stimulate tumor cells at the cell-cell contact sites and promote detachment and spread of individual cancer cells from neighboring tissue. Physical contact between Schwann cells and tumor cells is necessary to potentiate the process of tumor invasion, by induction of specific chemokines, such as CX3CL1 and its receptor CX3CL1 [2, 7, 8]. Schwann cells are also able to degrade the extracellular matrix, forming tunnels or bands coated with laminin. Tumor cells use these structures to migrate during tissue invasion. These activities strongly promote tumor spread and are dependent on NCAM1 expression by Schwann cells [2, 3, 7, 8].

Considering the intertwined structure of signaling, regulatory, and metabolic processes within a cell, we employed a genome-scale biomolecular network. Accordingly, a meta-analysis of Schwann cells associated transcriptomic datasets was performed, and the core information on differentially expressed genes (DEGs) was obtained by statistical analyses. Gene set over-representation analyses was performed on core DEGs to identify significantly functional and pathway enrichment analysis. DEGs were further integrated with genome-scale human biomolecular networks. miRNAs were proposed by the reconstruction of a transcriptional and post-transcriptional regulatory network. Moreover, microarray-based transcriptome profiling was performed, and the prognostic power of selected dedifferentiated Schwann cell biomolecules was predicted. Consequently, this systematic study reports candidate biomolecules that can be considered as biomarkers or potential targets for further experimental and clinical trials for perineural invasion risk in lung cancer patients.

## RESULTS

### Identification of differentially expressed genes, gene ontology enrichment, and functional classification

We obtained gene expression data belonging to specimens across two cancer types (LUAD and LUSC) from TCGA; these data were preprocessed using standard methods. After the identification of DEGs, a DAVID analysis was performed using them. Downregulated genes in both lung cancers belong to “complement and coagulation cascades” and “cell adhesion molecules (CAMs)” pathways. While “cell cycle” and “DNA replication” were the most active pathways in both lung cancers (Tables 1–4).

**Table 1:** Functional annotation analysis of downregulated differentially expressed genes (DEGs) in lung adenocarcinoma (LUAD) datasets using the DAVID tool

Term	Count	P Value	Genes	Pop Hits	Fold Enrichment
hsa04610:Complement and coagulation cascades	17	2,02E-05	F11, C7, A2M, F10, C5AR1, MASP1, C6, F8, SERPING1, C1QA, C8B, VWF, C1QB, THBD, CFD, PROS1, CPB2	69	3,423022096
hsa04270:Vascular smooth muscle contraction	21	1,10E-04	RAMP3, ADCY4, RAMP2, NPR1, PRKCE, PRKG1, MYL9, AGTR1, ACTG2, PRKCQ, PTGIR, GNAQ, ADCY9, GUCY1A2, PLA2G1B, MYH11, CALCRL, PLA2G3, PLA2G5, MYLK, PPP1R14A	112	2,605020492
hsa04514:Cell adhesion molecules (CAMs)	21	0,00103288	SELP, ITGAL, CLDN18, PTPRM, CADM1, ICAM2, CLDN5, NFASC, CLDN11, HLA-E, CDH5, SIGLEC1, CD34, ITGA8, ESAM, JAM2, JAM3, NEGR1, SELE, SELPLG, SPN	132	2,210320417
hsa04080:Neuroactive ligand-receptor interaction	32	0,00245857	F2RL3, THRB, LEPR, PTH1R, FPR1, GRIN3B, FPR2, VIPR1, APLNR, AGTR1, EDNRB, AGTR2, PTGIR, S1PR1, NMUR1, S1PR4, CNR1, P2RY1, S1PR5, CALCRL, GHR, GRID1, C5AR1, PTGER4, PTGFR, ADRB2, ADRB1, P2RX1, SSTR1, GRIA1, P2RY14, CTSG	256	1,736680328
hsa04614:Renin-angiotensin system	6	0,0055758	AGTR1, ACE, AGTR2, MME, CPA3, CTSG	17	4,903567985
hsa02010:ABC transporters	9	0,0117617	ABCA8, ABCC9, ABCA9, ABCB1, CFTR, ABCA3, ABCG1, ABCA6, ABCG2	44	2,841840537
hsa04670:Leukocyte transendothelial migration	16	0,02073563	ITGAL, CLDN18, NCF2, NCF1, CLDN5, CLDN11, CXCL12, CDH5, MYL9, CYBB, ESAM, PIK3R5, RAPGEF4, JAM2, JAM3, PIK3R1	118	1,883856627
hsa03320:PPAR signaling pathway	11	0,02420575	LPL, CD36, CYP27A1, SORBS1, OLR1, PPARG, RXRG, FABP4, SCD5, ACADL, FABP5	69	2,21489665
hsa04062:Chemokine signaling pathway	22	0,02578677	ADCY4, CCL2, FGR, NCF1, PREX1, HCK, CXCL3, CXCL2, CXCR1, GNG11, CXCR2, CXCL12, CCL14, CCL23, PPBP, ADCY9, ARRB1, RASGRP2, CX3CR1, PIK3R5, GRK5, PIK3R1	187	1,634522662
hsa04060:Cytokine-cytokine receptor interaction	28	0,03410983	CSF3, ACVRL1, CCL2, PDGFB, CXCL3, LEPR, CXCL2, BMPR2, CXCR1, CXCR2, TNFSF13, TNFSF12, IL7R, CXCL12, CCL23, PLEKHO2, FIGF, GHR, IL18R1, IL6, BMP2, FLT4, TGFBR2, LIFR, CCL14, PPBP, IL20RA, CX3CR1, IL3RA	262	1,484795395
hsa05020:Prion diseases	7	0,0361229	C1QA, EGR1, C1QB, C8B, C7, IL6, C6	35	2,778688525
hsa00590:Arachidonic acid metabolism	9	0,04474724	ALOX15, PTGIS, PTGDS, GPX3, PLA2G1B, LTC4S, ALOX5, PLA2G3, HPGDS, PLA2G5	56	2,232874707
hsa04350:TGF-beta signaling pathway	12	0,04546484	BMP2, SMAD9, ACVRL1, CDKN2B, ZFYVE9, SMAD6, LEFTY2, TGFBR2, BMPR2, ID4, DCN, ID3	87	1,916336914

The results revealed that downregulated consistent DEGs indicated the genes that were associated with the top four pathways: “Complement and coagulation cascades”, “Vascular smooth muscle contraction”, “Cell adhesion molecules (CAMs)” and, “Neuroactive ligand-receptor interaction”.

**Table 2:** Functional annotation analysis of upregulated differentially expressed genes (DEGs) in lung adenocarcinoma (LUAD) datasets using the DAVID tool

Term	Count	P Value	Genes	Pop Hits	Fold Enrichment
hsa04110:Cell cycle	43	2,82E-09	E2F1, E2F2, E2F3, E2F5, DBF4, PRKDC, TTK, PKMYT1, CHEK1, CHEK2, SFN, PTTG1, CCNE2, CCNE1, CDC45, MCM7, CDKN2A, ORC6L, BUB1, CCNA2, CDC7, CDC6, CDK1, RBL1, SKP2, ESPL1, CDC20, MCM2, CDC25C, MCM3, CDK4, MCM4, ORC1L, CDC25A, MCM6, CCNB1, MAD2L1, CCNB2, PLK1, PCNA, BUB1B, MAD2L2, SMC1B	125	2,591466667
hsa03030:DNA replication	17	4,23E-06	LIG1, POLE, MCM2, MCM3, RNASEH2A, MCM4, MCM6, RFC5, PRIM1, DNA2, RFC3, RFC4, MCM7, POLE2, PRIM2, PCNA, FEN1	36	3,557407407
hsa04512:ECM-receptor interaction	26	5,15E-05	IBSP, TNC, COL3A1, ITGA11, ITGB4, COL2A1, VTN, ITGB3, CHAD, HMMR, LAMB3, ITGB8, COMP, COL6A3, SV2A, COL11A2, COL11A1, THBS2, SPP1, THBS4, ITGA2, COL5A2, COL5A1, LAMA1, COL1A2, COL1A1	84	2,331746032
hsa00270:Cysteine and methionine metabolism	13	9,00E-04	DNMT3A, LDHB, LDHA, AHCY, SRM, IL4I1, CTH, GOT1, SDS, MAT1A, DNMT1, DNMT3B, CBS	34	2,880392157
hsa00330:Arginine and proline metabolism	17	9,64E-04	PYCRL, ODC1, ALDH18A1, CKMT1B, SRM, AGMAT, CPS1, PYCR1, ABP1, CKMT1A, GOT1, CKM, ALDH1B1, P4HA1, P4HA3, GAMT, OAT, ADC	53	2,416352201
hsa00250:Alanine, aspartate and glutamate metabolism	12	0,00139536	ADSSL1, GOT1, ACY3, GFPT1, IL4I1, GPT, CAD, ASNS, CPS1, GAD1, GPT2, PPAT	31	2,916129032
hsa00010:Glycolysis / Gluconeogenesis	18	0,00148413	ALDOA, LDHB, LDHA, ALDOB, PFKP, ADH1C, ALDH3B2, PGAM2, PCK1, ALDH3A1, GPI, TPI1, G6PC, ALDH1B1, PKM2, ENO3, GAPDH, ENO1	60	2,26
hsa00601:Glycosphingolipid biosynthesis	10	0,0033785	B4GALT3, B4GALT2, FUT9, B3GALT5, B3GNT4, FUT6, FUT3, B3GNT3, FUT2, B4GALT4	25	3,013333333
hsa04115:p53 signaling pathway	18	0,00619724	CDK1, LRDD, CHEK1, CHEK2, PMAIP1, SFN, CDK4, GTSE1, CCNB1, CCNE2, CCNE1, CCNB2, CDKN2A, SERPINB5, RRM2, BAI1, PERP, IGFBP3	68	1,994117647
hsa00051:Fructose and mannose metabolism	11	0,01033738	ALDOA, TPI1, AKR1B15, SORD, GMDS, AKR1B10, GMPPA, ALDOB, PFKP, TSTA3, MTMR7, PMM2	34	2,437254902
hsa04950:Maturity onset diabetes of the young	9	0,0124429	HNF1A, HNF4A, BHLHA15, FOXA3, MNX1, NEUROD1, IGF2, HNF4G, PDX1	25	2,712
hsa00260:Glycine, serine and threonine metabolism	10	0,01604608	CTH, SHMT2, SDS, GCAT, DMGDH, GAMT, PSAT1, PSPH, CBS, GLDC	31	2,430107527
hsa03440:Homologous recombination	9	0,02497133	XRCC3, XRCC2, BLM, EME1, SHFM1, BRCA2, RAD54B, RAD54L, RAD51	28	2,421428571
hsa00533:Keratan sulfate biosynthesis	6	0,02885381	B4GALT3, B4GALT2, FUT8, CHST6, CHST4, B4GALT4	14	3,228571429
hsa00140:Steroid hormone biosynthesis	12	0,0343766	AKR1C3, AKR1C2, UGT1A6, UGT1A10, UGT1A9, AKR1C4, HSD17B2, HSD17B1, CYP11B1, SRD5A3, UGT2B4, SRD5A1, UGT2B15, AKR1C1	46	1,965217391
hsa00512:O-Glycan biosynthesis	9	0,03707018	GALNT3, GCNT3, GALNT7, GALNT6, GALNT4, B3GNT6, C1GALT1, GALNT14, ST6GALNAC1	30	2,26
hsa05219:Bladder cancer	11	0,04377571	E2F1, E2F2, E2F3, CDKN2A, PGF, MMP9, ERBB2, CDH1, EGF, CDK4, MMP1	42	1,973015873
hsa00830:Retinol metabolism	13	0,04676524	BCMO1, CYP2C18, CYP2B6, ADH1C, RDH5, UGT1A10, UGT1A6, UGT1A9, RDH10, LRAT, DGAT1, DGAT2, CYP2A6, UGT2B4, UGT2B15	54	1,813580247
hsa00980:Metabolism of xenobiotics by cytochrome P450	14	0,04737766	CYP2C18, CYP2B6, ADH1C, ALDH3B2, CYP2E1, ALDH3A1, AKR1C3, UGT1A10, UGT1A6, AKR1C2, UGT1A9, AKR1C4, UGT2B4, UGT2B15, AKR1C1, MGST1	60	1,757777778

The results revealed that upregulated consistent DEGs indicated the genes that were associated with the top three pathways: “Cell cycle”, “DNA replication” and, “ECM-receptor interaction”.

**Table 3:** Functional annotation analysis of downregulated differentially expressed genes (DEGs) in Lung squamous cell carcinoma (LUSC) datasets using the DAVID tool

Term	Count	P Value	Genes	Pop Hits	Fold Enrichment
hsa04610:Complement and coagulation cascades	31	1,15E-10	C3AR1, C7, A2M, MASP1, C3, F13A1, C6, C5, C1QC, FGG, SERPINA1, C2, CFI, CFD, F11, CR1, F10, C5AR1, C4A, F8, SERPING1, C4BPA, C1QA, C1QB, VWF, C8B, CD55, TFPI, SERPIND1, CPB2, PROS1	69	3,586444611
hsa04514:Cell adhesion molecules (CAMs)	44	8,51E-10	HLA-DQB1, ITGAL, CADM3, CLDN18, CADM1, HLA-DRB1, CLDN5, ITGB2, HLA-DMB, SDC4, HLA-DMA, CDH5, ITGAM, CD22, HLA-DRB5, ESAM, CD4, HLA-DPB1, HLA-DOA, SELPLG, NEGR1, SPN, ICAM1, SELP, PTPRC, PTPRM, ICAM2, HLA-E, HLA-DQA2, CLDN23, HLA-DQA1, SIGLEC1, NCAM2, ITGA9, CD86, CD34, ITGA8, CLDN2, HLA-DPA1, JAM2, JAM3, SELE, CD226, HLA-DRA	132	2,660910518
hsa04640:Hematopoietic cell lineage	30	2,22E-07	CSF3, IL1R1, HLA-DRB1, CSF1, MME, ANPEP, IL7R, ITGAM, FCGR1C, FCGR1A, CSF3R, CD22, HLA-DRB5, CD4, CSF2RA, CSF1R, IL6, CR1, CD1C, ITGA1, IL6R, IL11RA, CD1E, CD55, CD37, CD36, CD34, CD33, EPOR, IL3RA, HLA-DRA	86	2,784673798
hsa05416:Viral myocarditis	25	2,37E-06	HLA-DQB1, PRF1, ITGAL, CAV1, HLA-DRB1, ITGB2, HLA-DMB, HLA-DMA, HLA-DRB5, HLA-DPB1, HLA-DOA, ICAM1, HLA-E, HLA-DQA2, HLA-DQA1, LAMA2, CD86, CD55, SGCG, MYH11, SGCD, HLA-DPA1, SGCA, HLA-DRA, MYH10	71	2,81082097
hsa04060:Cytokine-cytokine receptor interaction	58	1,18E-05	ACVRL1, PDGFB, IL6ST, LEPR, TNFSF15, CXCR1, CXCR2, TNFSF13, TNFSF12, CXCL12, TGFB2, CSF3R, CSF2RB, CSF2RA, IL18RAP, LIFR, IL6R, IL11RA, TNFRSF10C, PPBP, TNFRSF10D, CCR2, CX3CR1, TNFSF12-TNFSF13, CSF3, CCL3, IL1R1, CCL2, CXCL5, CSF1, CCR1, CXCL3, CXCL2, BMPR2, IL7R, CCL4, LIF, TNFRSF1B, IL12RB1, CCL21, IL10RA, PLEKHO2, FIGF, CSF1R, IL18R1, IL6, BMP2, FLT1, FLT4, TGFBR2, EDA2R, HGF, CCL18, KDR, CCL17, CCL13, CCL14, CXCL16, EPOR, IL3RA	262	1,767169581
hsa04062:Chemokine signaling pathway	45	1,61E-05	ADCY4, PRKCZ, CCL3, CCL2, GNAI2, CXCL5, FGR, PREX1, CXCL3, CCR1, CXCL2, NCF1C, NFKBIA, CXCR1, GNG11, CXCR2, CCL4, CXCL12, DOCK2, CCL21, RASGRP2, GNG2, PIK3R5, SHC3, PLCB2, GNG7, PIK3CG, ITK, NCF1, HCK, WAS, CCL18, ELMO1, PRKCB, CCL17, GNGT2, CCL13, CCL14, PPBP, ADCY9, ARRB2, ARRB1, CXCL16, CCR2, CX3CR1, GRK5	187	1,920978181
hsa05310:Asthma	14	1,62E-05	FCER1A, HLA-DQB1, HLA-DRB1, HLA-DMB, HLA-DMA, HLA-DQA2, HLA-DQA1, HLA-DRB5, MS4A2, FCER1G, HLA-DPA1, HLA-DPB1, HLA-DOA, HLA-DRA	29	3,853732474
hsa04270:Vascular smooth muscle contraction	31	2,82E-05	ADCY4, ADORA2A, PPP1R12B, MRVI1, PRKG1, MYL9, EDNRA, AGTR1, ACTG2, PTGIR, GUCY1A2, PLA2G1B, GUCY1A3, CALCRL, PLCB2, PPP1R14A, RAMP3, RAMP2, PLA2G10, NPR1, PRKCE, ITPR1, PRKCB, PRKCQ, ADCY9, GNAQ, MYH11, CACNA1C, CACNA1D, PLA2G5, MYLK	112	2,209506055
hsa05332:Graft-versus-host disease	16	3,38E-05	HLA-DQB1, PRF1, IL6, HLA-DRB1, HLA-DMB, HLA-E, HLA-DMA, HLA-DQA2, HLA-DQA1, CD86, HLA-DRB5, HLA-DPA1, HLA-DPB1, HLA-DOA, KLRD1, HLA-DRA	39	3,274966791
hsa05322:Systemic lupus erythematosus	27	1,37E-04	HLA-DQB1, C7, HLA-DRB1, C3, C6, C5, HLA-DMB, C1QC, HLA-DMA, FCGR1C, FCGR1A, HLA-DRB5, HLA-DPB1, C2, FCGR3A, HLA-DOA, FCGR3B, C4A, HLA-DQA2, HLA-DQA1, C1QA, C8B, C1QB, CD86, HLA-DPA1, FCGR2A, CTSG, HLA-DRA	99	2,177108606
hsa04672:Intestinal immune network for IgA production	17	1,84E-04	HLA-DQB1, IL6, HLA-DRB1, TNFSF13, TNFSF12, PIGR, HLA-DMB, HLA-DMA, CXCL12, HLA-DQA2, HLA-DQA1, TGFB2, CD86, HLA-DRB5, HLA-DPA1, HLA-DPB1, TNFSF12-TNFSF13, HLA-DOA, HLA-DRA	49	2,769519111
hsa05330:Allograft rejection	14	2,39E-04	HLA-DQB1, PRF1, HLA-DRB1, HLA-DMB, HLA-E, HLA-DMA, HLA-DQA2, HLA-DQA1, CD86, HLA-DRB5, HLA-DPA1, HLA-DPB1, HLA-DOA, HLA-DRA	36	3,104395604
hsa04940:Type I diabetes mellitus	15	3,63E-04	HLA-DQB1, PRF1, HLA-DRB1, PTPRN2, HLA-DMB, HLA-E, HLA-DMA, HLA-DQA2, HLA-DQA1, CD86, HLA-DRB5, HLA-DPA1, HLA-DPB1, HLA-DOA, HLA-DRA	42	2,850975555
hsa05414:Dilated cardiomyopathy	24	7,06E-04	ADCY4, SLC8A1, TNNC1, ITGA1, CACNG4, ITGA10, CACNB4, TTN, CACNA2D2, TGFB2, LAMA2, ITGA9, DES, ADRB1, SGCG, ADCY9, PLN, ITGA8, ITGA7, RYR2, SGCD, CACNA1C, CACNA1D, SGCA	92	2,08245171
hsa05410:Hypertrophic cardiomyopathy (HCM)	22	0,00139878	SLC8A1, IL6, TNNC1, ITGA1, CACNG4, ITGA10, CACNB4, TTN, CACNA2D2, TGFB2, LAMA2, ITGA9, ACE, DES, SGCG, ITGA8, ITGA7, RYR2, SGCD, CACNA1C, CACNA1D, SGCA	85	2,066118755
hsa04614:Renin-angiotensin system	8	0,00290177	AGTR1, ACE, AGTR2, MME, CPA3, ANPEP, ENPEP, CTSG	17	3,756579555
hsa05320:Autoimmune thyroid disease	14	0,0083188	HLA-DQB1, PRF1, HLA-DRB1, HLA-DMB, HLA-E, HLA-DMA, HLA-DQA2, HLA-DQA1, CD86, HLA-DRB5, HLA-DPA1, HLA-DPB1, HLA-DOA, HLA-DRA	51	2,191338074
hsa04670:Leukocyte transendothelial migration	25	0,00994356	ITGAL, CLDN18, GNAI2, CLDN5, NCF1C, ITGB2, CXCL12, CDH5, ITGAM, MYL9, ESAM, PIK3R5, RAPGEF4, RAPGEF3, PIK3CG, ICAM1, ITK, NCF2, NCF1, NCF4, CLDN23, PRKCB, CYBB, CLDN2, JAM2, JAM3	118	1,691256685
hsa04612:Antigen processing and presentation	19	0,01258302	HLA-DQB1, CIITA, HLA-DRB1, IFI30, CTSS, HLA-DMB, HLA-E, HLA-DMA, HLA-DQA2, HLA-DQA1, CD74, B2M, HLA-DRB5, CD4, HLA-DPA1, HLA-DPB1, HLA-DOA, KLRD1, HLA-DRA	83	1,827372283
hsa05412:Arrhythmogenic right ventricular cardiomyopathy (ARVC)	17	0,02357362	SLC8A1, ITGA1, CACNG4, ITGA10, CACNB4, CACNA2D2, LAMA2, ITGA9, DES, SGCG, ITGA8, ITGA7, RYR2, SGCD, CACNA1C, CACNA1D, SGCA	76	1,785611006
hsa04530:Tight junction	26	0,02458398	PRKCZ, CLDN18, GNAI2, MRAS, CLDN5, MYL9, RRAS, PARD6B, MAGI3, MAGI1, MPDZ, PRKCE, CLDN23, PRKCB, EPB41L2, EPB41L3, PRKCQ, TJP1, CGN, MYH11, CLDN2, TJP3, JAM2, JAM3, MYH10, SPTAN1	134	1,548888212
hsa05020:Prion diseases	10	0,02477695	C1QA, EGR1, C1QB, NCAM2, C8B, C7, IL6, C6, C5, C1QC	35	2,280780444
hsa04960:Aldosterone-regulated sodium reabsorption	11	0,0263487	PIK3CG, ATP1B2, HSD11B1, NR3C2, PIK3R5, NEDD4L, ATP1A2, SCNN1G, SCNN1B, SLC9A3R2, PRKCB	41	2,141708466
hsa03320:PPAR signaling pathway	15	0,04364393	ACOX2, LPL, OLR1, PPARG, RXRG, ACADL, ACSL1, CD36, SORBS1, CYP27A1, HMGCS2, FABP3, FABP4, ACSL4, ACSL5	69	1,735376425
hsa04666:Fc gamma R-mediated phagocytosis	19	0,04492025	PIK3CG, DNM3, PTPRC, WASF3, NCF1, HCK, NCF1C, PIP5K1B, PRKCE, WAS, PRKCB, DOCK2, GAB2, FCGR1C, CFL2, FCGR1A, PLA2G4F, PIK3R5, FCGR2A, FCGR3A, PPAP2B	95	1,596546311
hsa04020:Calcium signaling pathway	31	0,04546262	GNA14, ADCY4, ERBB4, CYSLTR1, ADORA2A, TNNC1, EDNRA, AGTR1, EDNRB, PDE1B, PLCB2, SLC8A1, BST1, PTGFR, ITPR1, PRKCB, ADRB2, P2RX7, PLCE1, ADRB1, P2RX1, ADCY9, GNAQ, PLN, TBXA2R, CACNA1H, RYR2, CACNA1C, CACNA1D, MYLK, PTAFR	176	1,406049308

The results revealed that downregulated consistent DEGs indicated the genes that were associated with the top three pathways: “Complement and coagulation cascades”, “Cell adhesion molecules (CAMs)” and, “Hematopoietic cell lineage”.

**Table 4:** Functional annotation analysis of upregulated differentially expressed genes (DEGs) in Lung squamous cell carcinoma (LUSC) datasets using the DAVID tool

Term	Count	P Value	Genes	Pop Hits	Fold Enrichment
hsa04110:Cell cycle	54	7,45E-13	E2F1, E2F2, E2F3, DBF4, PKMYT1, TTK, PTTG1, CCNE2, CCNE1, CDC45, MCM7, CDKN2A, CCNA1, CCNA2, CDC7, CDK1, CDC6, RBL1, SKP2, CDK6, ESPL1, MCM2, MCM3, CDK4, MCM4, MCM5, ORC1L, CDK2, MCM6, MAD2L1, BUB1B, ORC5L, ANAPC7, MAD2L2, YWHAZ, PRKDC, CHEK1, CHEK2, SFN, ORC6L, BUB1, TFDP2, TFDP1, CDC20, ATR, CDC25C, CDC25A, CCNB1, HDAC2, CCNB2, HDAC1, PLK1, PCNA, SMC1B	125	2,715352287
hsa03030:DNA replication	25	9,11E-12	RNASEH1, POLA2, RPA3, PRIM1, MCM7, POLE2, POLE3, PRIM2, FEN1, LIG1, POLE, MCM2, RNASEH2A, MCM3, MCM4, MCM5, MCM6, RFC5, DNA2, RFC3, RFC4, RFC2, POLD1, POLD2, PCNA	36	4,364956737
hsa04115:p53 signaling pathway	27	6,14E-06	BID, LRDD, RPRM, CHEK1, SFN, CHEK2, PMAIP1, GTSE1, SESN3, CCNE2, CCNE1, CDKN2A, BAI1, TP53AIP1, CDK1, CYCS, CDK6, ATR, CDK4, CDK2, TP73, CCNB1, CCNB2, SERPINB5, RRM2, PERP, IGFBP3	68	2,495728205
hsa00480:Glutathione metabolism	21	3,38E-05	GSTA1, ODC1, GCLC, GGCT, PGD, GCLM, GSS, GSTM1, GPX2, GSTM2, GSR, GSTM3, GSTM4, G6PD, OPLAH, RRM2, RRM1, IDH2, GSTO2, GPX7, SMS	50	2,639925834
hsa03410:Base excision repair	16	1,29E-04	APEX2, UNG, NEIL3, LIG1, POLE, LIG3, POLB, SMUG1, POLE2, POLE3, POLD1, POLD2, PCNA, TDG, PARP1, FEN1	35	2,873388663
hsa05217:Basal cell carcinoma	21	1,64E-04	FZD9, WNT5A, DVL3, WNT10A, WNT16, WNT10B, WNT5B, LEF1, FZD3, GLI2, FZD7, FZD6, GLI1, WNT2B, SMO, FZD10, WNT7B, WNT4, WNT3, WNT11, PTCH2	55	2,399932577
hsa03430:Mismatch repair	12	3,26E-04	RFC5, EXO1, MSH6, RFC3, RFC4, RFC2, MSH2, POLD1, LIG1, POLD2, PCNA, RPA3	23	3,279410974
hsa00010:Glycolysis / Gluconeogenesis	21	6,17E-04	ALDOA, LDHA, ALDOC, PGAM1, HK2, PFKP, ADH1C, ALDH3B2, PGAM2, ADH7, PCK1, ALDH3A1, PGM2, GPI, TPI1, PKM2, ENO2, ENO3, PGK1, GAPDH, ENO1	60	2,199938195
hsa05200:Pathways in cancer	75	6,70E-04	FGF19, E2F1, E2F2, E2F3, HRAS, PGF, MMP9, FGF11, FGF12, GLI2, MMP1, ACVR1C, GLI1, CCNE2, CCNE1, WNT4, WNT3, CDKN2A, SLC2A1, TGFA, NOS2, CCNA1, FGF3, EGFR, WNT10A, RET, PLD1, WNT10B, CYCS, SKP2, LEF1, FADD, CDK6, CDK4, CDK2, RAD51, JUP, SMO, PIAS3, LAMC2, WNT11, WNT5A, BID, CKS1B, WNT16, WNT5B, FGFR3, EGLN3, TFG, CDH1, LAMB3, RAC3, EGF, TRAF4, PIK3R2, FZD9, MSH6, DVL3, MSH2, BRCA2, ITGA2, FZD3, BIRC5, COL4A6, FZD7, FZD6, WNT2B, LAMA1, CBLC, FZD10, WNT7B, HDAC2, ITGA6, HDAC1, PTCH2	328	1,437241852
hsa04114:Oocyte meiosis	32	6,81E-04	YWHAZ, ADCY2, PKMYT1, AURKA, PTTG1, PRKX, CCNE2, CCNE1, CALML3, FBXO43, BUB1, STAG3, FBXO5, CAMK2B, CALML5, CDK1, SGOL1, CHP2, IGF2, ESPL1, CDC20, CDC25C, CDK2, CCNB1, RPS6KA6, MAD2L1, CCNB2, MAPK12, PLK1, ANAPC7, MAD2L2, SMC1B	110	1,828520058
hsa00240:Pyrimidine metabolism	28	0,00129063	POLR2H, CTPS, PNPT1, DTYMK, CAD, POLA2, POLR2D, TK1, PRIM1, TYMS, POLE2, NT5M, POLE3, PRIM2, ENTPD3, UCK2, POLR3G, POLE, POLR1C, POLR2J3, NME4, UMPS, NME2, NME1-NME2, NME1, RRM2, POLD1, POLD2, RRM1, TXNRD1	95	1,852579533
hsa00250:Alanine, aspartate and glutamate metabolism	13	0,00179493	GLS2, GOT2, ADSSL1, ALDH4A1, ADSL, IL4I1, GPT, CAD, ASNS, CPS1, GAD1, GPT2, PPAT	31	2,635870649
hsa04340:Hedgehog signaling pathway	19	0,00182644	WNT5A, WNT10A, WNT16, WNT10B, WNT5B, GLI2, PRKX, ZIC2, GLI1, WNT2B, SMO, WNT7B, WNT4, WNT3, WNT11, PTCH2, BMP7, BMP8B, BMP8A	56	2,132593149
hsa04512:ECM-receptor interaction	25	0,0021767	TNC, COL3A1, ITGB4, ITGA11, ITGA2, COL2A1, COL5A2, COL5A1, COL4A6, HMMR, LAMA1, LAMB3, SDC1, ITGA6, ITGB8, COMP, COL1A2, LAMC2, SV2B, SV2A, COL1A1, COL11A2, THBS2, COL11A1, SPP1	84	1,870695744
hsa03440:Homologous recombination	12	0,00241416	XRCC3, XRCC2, BLM, POLD1, POLD2, EME1, SHFM1, BRCA2, RAD54B, RAD54L, RAD51, RPA3	28	2,693801872
hsa00360:Phenylalanine metabolism	10	0,00433744	GOT2, DDC, NAA20, LCLAT1, ALDH3B2, IL4I1, PAH, PNPLA3, MIF, ALDH3A1	22	2,857062591
hsa00230:Purine metabolism	37	0,00801809	XDH, POLR2H, GDA, ADCY2, PNPT1, POLA2, POLR2D, HPRT1, AK3L1, ADA, PPAT, PRIM1, POLE2, ATIC, POLE3, NT5M, PRIM2, ENTPD3, ADCY10, ENTPD2, POLR3G, ADSSL1, POLE, POLR1C, GMPS, POLR2J3, GART, NME4, NME2, NME1-NME2, NME1, RRM2, POLD1, PKM2, POLD2, RRM1, ADSL, PAICS, PRPS2	153	1,520031993
hsa00980:Metabolism of xenobiotics by cytochrome P450	18	0,01003269	GSTA1, CYP2C18, CYP2S1, ADH1C, ALDH3B2, ADH7, CYP2E1, UGT1A1, ALDH3A1, AKR1C3, GSTM1, UGT1A7, GSTM2, AKR1C2, UGT1A10, UGT1A6, UGT1A9, GSTM3, GSTM4, UGT1A8, UGT2A1, GSTO2, AKR1C1	60	1,88566131
hsa00030:Pentose phosphate pathway	10	0,01140598	PGM2, ALDOA, GPI, TALDO1, G6PD, ALDOC, PGD, PFKP, TKTL1, PRPS2	25	2,51421508
hsa00330:Arginine and proline metabolism	16	0,01525979	PYCRL, ODC1, ALDH18A1, CKMT1B, AGMAT, CPS1, GLS2, GOT2, ABP1, PYCR1, CKMT1A, P4HA1, P4HA3, ALDH4A1, GAMT, NOS2, SMS	53	1,897520815
hsa04310:Wnt signaling pathway	35	0,0189717	WNT5A, WNT16, WNT5B, MMP7, PRKX, WNT4, CSNK2A1, WNT3, RAC3, CACYBP, CAMK2B, FZD9, WNT10A, TBL1XR1, DVL3, WNT10B, VANGL1, VANGL2, CHP2, LEF1, FZD3, CSNK2A1P, PORCN, FZD7, WNT2B, FZD6, DKK4, SENP2, WNT7B, FZD10, DKK1, SFRP1, SFRP2, WNT11, RUVBL1, TBL1X	151	1,456912712
hsa04916: Melanogenesis	25	0,01931079	WNT5A, HRAS, WNT16, ADCY2, WNT5B, POMC, PRKX, WNT4, WNT3, MC1R, CALML3, CAMK2B, CALML5, TUBB3, FZD9, DVL3, WNT10A, WNT10B, LEF1, FZD3, FZD7, FZD6, WNT2B, WNT7B, FZD10, WNT11	99	1,587256995
hsa05219:Bladder cancer	13	0,02646666	E2F1, EGFR, E2F2, E2F3, HRAS, FGFR3, PGF, MMP9, CDH1, CDK4, MMP1, CDKN2A, EGF	42	1,945523574
hsa00670:One carbon pool by folate	7	0,0303469	TYMS, MTHFD2, SHMT2, ALDH1L1, DHFR, ATIC, GART	16	2,749922744
hsa05222:Small cell lung cancer	21	0,03694956	E2F1, E2F2, CKS1B, E2F3, CYCS, SKP2, ITGA2, CDK6, CDK4, CDK2, COL4A6, CCNE2, LAMA1, CCNE1, LAMB3, ITGA6, PIAS3, LAMC2, NOS2, TRAF4, PIK3R2	84	1,571384425

The results revealed that upregulated consistent DEGs indicated the genes that were associated with the top three pathways: “Cell cycle”, “DNA replication” and, “p53 signaling pathway”.

As Schwann cells act on both of the aforementioned upregulated pathways, we selected datasets from the GEO database containing gene expression profiles of both early and late passage human Schwann cells exposed to cancer growth factors heregulin and forskolin (GSE4030). These expression profiles were used to identify DEGs with the aid of the online tool GEO2R. We found 638 upregulated and 1250 downregulated genes. Then, we performed a GO term enrichment and functional classification by KEGG analysis, using DAVID platform, to investigate the biological and functional roles of these DEGs. Upregulated DEG enrichment included “neuroactive ligand-receptor interaction,” while “pathways in cancer” and “focal adhesion” were enriched for downregulated DEGs (Supplementary Tables 1-2).

**Figure 1:**
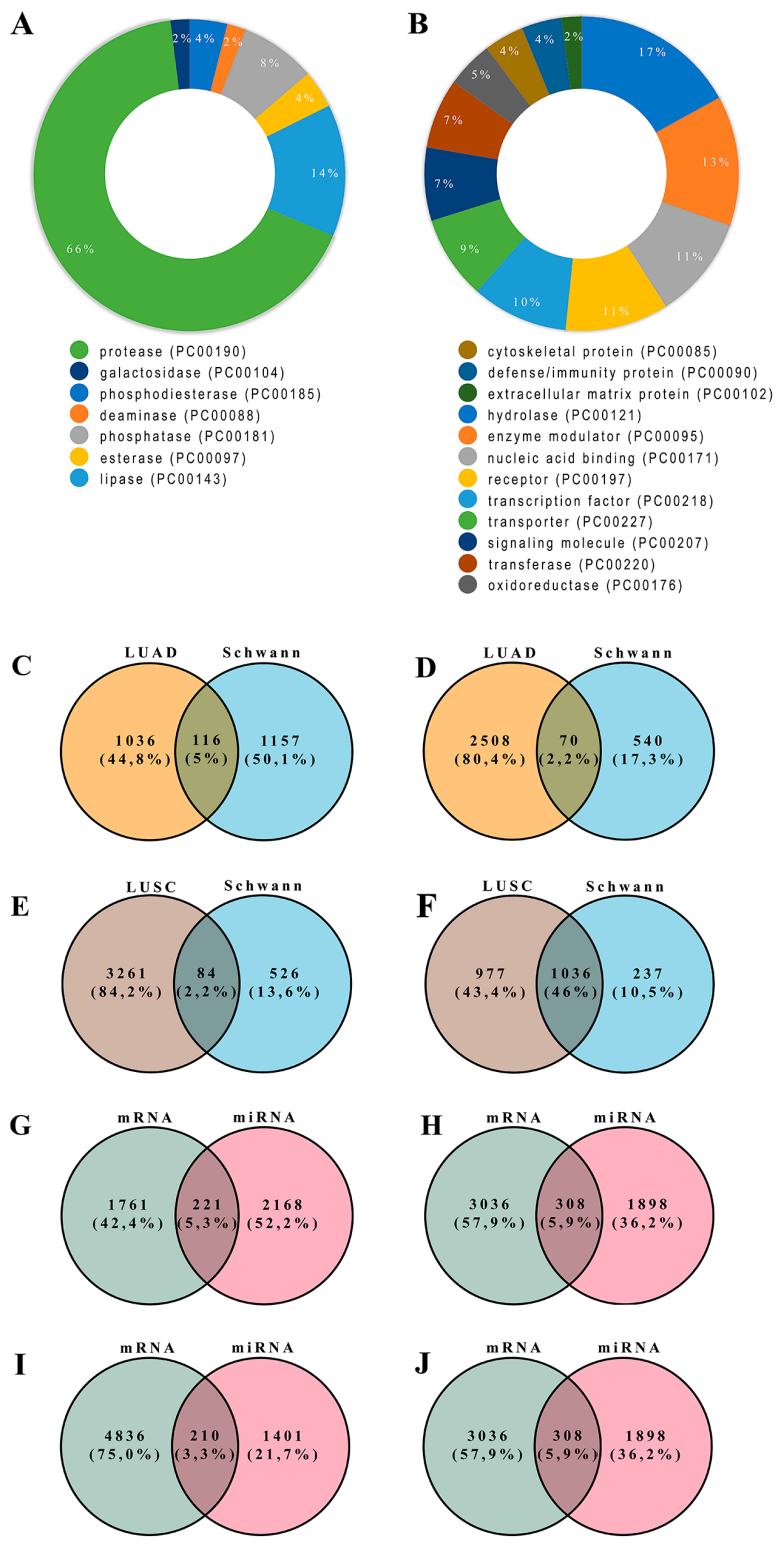
Molecular function and protein class terms of downregulated. **(A)** and upregulated **(B)** among the differentially regulated genes in Schwann cells. Figure 1C-D - Venn Diagrams of combined overrepresented differentially expressed genes in Schwann cells and lung adenocarcinoma **(C and D)**, and Lung squamous cell carcinoma **(E and F)** cancer samples. The results are showing overlapping downregulated (Figure 1C) and upregulated (Figure 1D) genes between lung adenocarcinoma and Schwan cells. Overlapped downregulated and upregulated genes between Lung squamous cell carcinoma and Schwan cells are showed in Figure 1E and Figure 1F, respectively. Figure 1G-H - Venn Diagrams of downregulated miRNA target genes combined with upregulated genes in lung adenocarcinoma **(G)** and Lung squamous cell carcinoma **(H)**. Figure 1I-J - Venn Diagrams of upregulated miRNA target genes combined with downregulated genes in lung adenocarcinoma **(I)** and Lung squamous cell carcinoma **(J)**. The number in each intersecting region represents the number of overlapping genes.

To facilitate the analysis of the large throughput of DEGs, a protein classification analysis was performed using the PANTHER classification system. According to the study, upregulated genes categories were identified, in which hydrolase (PC00121), enzyme modulator (PC00095), nucleic acid binding (PC00171), receptor (PC00197), transcription factor (PC00218), transporter (PC00227), and signaling molecule (PC00207) are the top seven most abundant protein classes. However, protease (PC00190) is the top one most abundant protein class among downregulated DEGs (Figure 1A-B).

### Overview of the cancer transcriptomic analysis

We conducted a systematic and integrative analysis to explore cancer type-specific and Schwann cell specific DEGs, to construct a cancer network. We first determined DEGs by comparing gene expression levels between tumor and normal samples. A Venn diagram was then constructed to visualize the overlap between DEG genes, both upregulated and downregulated, from both cancer types and from Schwann cells. Results are shown in Figure 1C-F. Among upregulated genes from LUSC and LUAD, we identified 84 and 70 co-expressed DEGs with Schwann cells, respectively.

We also ran a KEGG analysis to investigate pathways with major expression changes in studied cell lines and lung cancer, based on previously overlapping identified DEGs. The analysis revealed that “neuroactive ligand-receptor interaction” and “p53 signaling pathway” were among the most active pathways according to overlapped Schwann cells and LUSC DEGs. In contrast, overlapped Schwann cells and LUAD DEGs samples show “neuroactive ligand-receptor interaction” pathway as mainly downregulated, while “p53 signaling pathway” is upregulated (Supplementary Tables 3-6). *Grm1*, *Chrm3*, *Gabra5*, *Gpr50*, and *Pth2r* were the common upregulated genes associated with the “neuroactive ligand-receptor interaction” pathway; *Ccne1*, *Cdkn2a*, and *Perp* were commonly associated to “p53 signaling pathway”.

To confirm our results, we performed a differentially expressed miRNA analysis over cancer data. Data from identified miRNAs target genes were crossed with DEGs data from Schwann cells (Figure 1G-J). We found that downregulated miRNAs had target genes associated with “neuroactive ligand-receptor interaction” in LUAD and “pathways in cancer” in both cancers (Supplementary Tables 7-8). In contrast, upregulated miRNAs had target genes associated with the “axon guidance” pathway in both lung cancers (Supplementary Tables 7-10). Genes from the “axon guidance” pathway, common to all our analyses, were *Robo2* and *Slit2* genes. ROBO2 and SLIT2 proteins were highly expressed in normal tissue compared to cancer samples (data not shown).

### Hallmarks of cancer analysis

In order to understand the mechanism by which Schwann cells aid in neoplastic development, we analyzed the behavior of genes associated with cell differentiation processes (*Gata3*, *Cdh1*, and *Cdh2*), apoptosis (*Casp3*, *Casp9*, *Bax*, and *Bcl2*), motility (*Cxcr2*, *Cxcl5*, *Mmp9*, and *Ccl12*), and cell proliferation (*Mki67*). While *Cdh1* and *Cdh2* showed an increased mRNA expression in LUSC samples, only CDH2 protein expression was decreased in LUAD when compared with LUSC (data not shown). Also, *Mmp9* and *Mki67* were overexpressed in both lung cancers (Supplementary Figures 1-2).

### Analysis of genes involved in dedifferentiation of Schwann cells

Schwann cells produce cell differentiation maintenance proteins (*Sox10*, *S100*, *Egr2*, *Mbp*, and *Mpz*) that, after nerve damage, diminish their expression, provoking cellular dedifferentiation. Consequently, the expression of a new set of proteins that form non-myelinated cells, *Sox10*, *Gap43*, *S100*, *Ncam1*, *Ngfr1*, and *Gfap*, is augmented. We observed increased expression of *Gap43* and *Gfap* genes in both lung cancers, whereas *Ngfr1* was upregulated only in LUSC cells (Supplementary Figures 1-2).

### Methylation and protein analyses

Methylation analysis of the *Cdh1*, *Cdh2*, *Mmp9*, *Mki67*, *Gfap*, *Gap43*, *Robo2*, and *Slit2* genes from tumor samples demonstrated that all of them were methylated in their promoter regions unlike those from normal tissues. However, there was no significant correlation between methylation and *Gfap* gene expression in lung cancers. Whereas there was a positive correlation between methylation and *Gap43* gene expression in LUSC samples (Supplementary Figures 3-24).

### Copy number alteration

**Figure 2:**
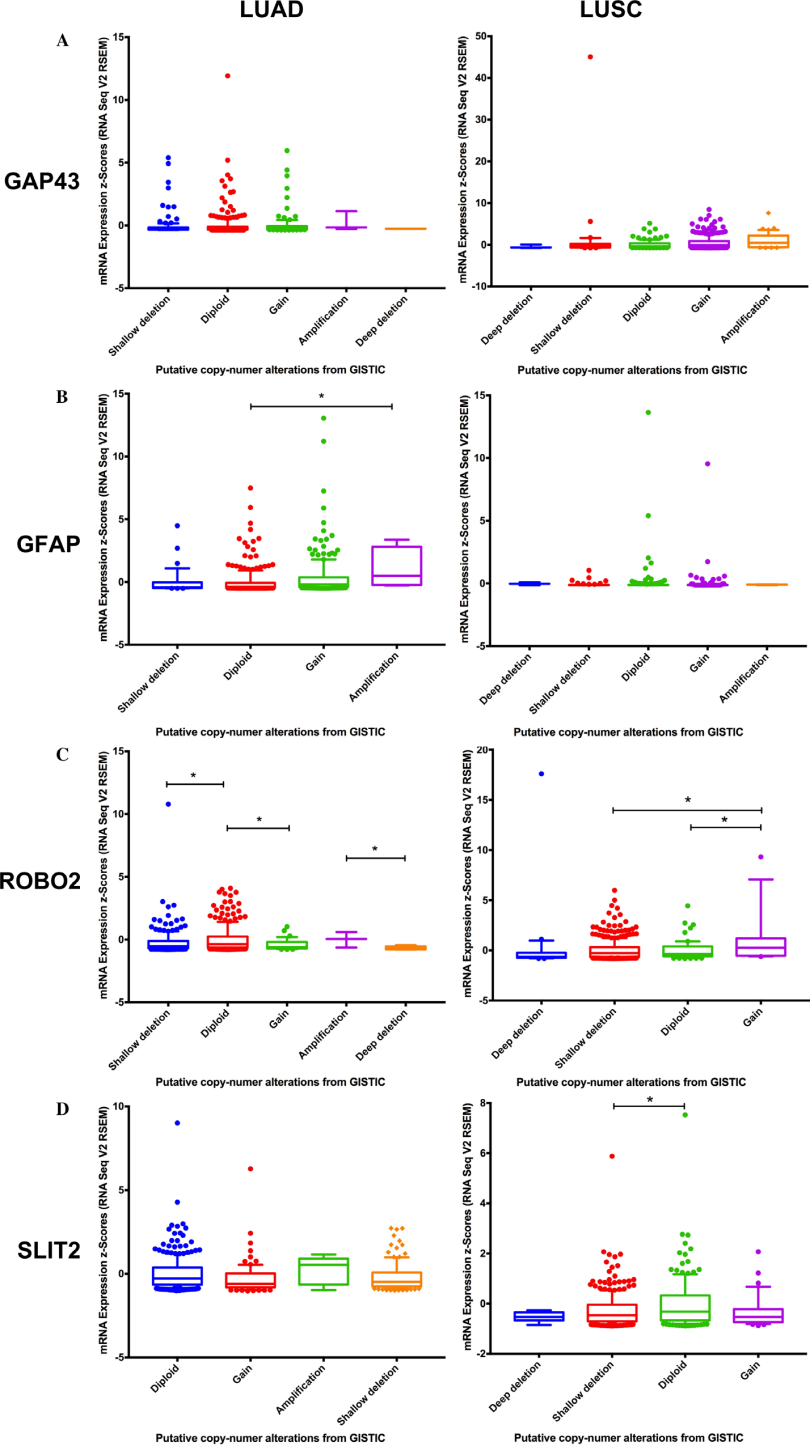
Correlation of copy number variation and expression of Gap43 **(A)**, Gfap **(B)**, Robo2 **(C)** and Slit2 **(D)** genes in lung adenocarcinom a (LUAD) and lung squamous cell carcinoma (LUSC). Figures were generated using Cbioportal data.

Copy number alteration data demonstrated that *Cdh1*, *Cdh2*, *Gfap*, *Perp*, and *Robo2* had a higher mRNA expression than normal tissues; increased expression was associated with gain or amplification alterations in LUAD samples (Supplementary Figure 25). Similarly, in LUSC samples, *Ccne1*, *Cdh1*, *Cdkn2a*, *Gap43*, and *Perp* had a higher mRNA expression associated with gain or amplification alterations (Figure 2).

### Schwann cell differentiation protein expression in lung cancer samples

For analysis of proteins expressed in dedifferentiated Schwann cells, we initially identified the gene list in Pubmed and GeneCards databases. We only analyzed genes with relevance score higher than seven. The relevance scores of elements related to genes are based on the analysis of co-occurrences of two elements in Medline documents. The observed are compared to an expected value based on a hypergeometric distribution. We identified 325 Schwann cell dedifferentiated related genes in both databases. Data were then cross-checked with previously published protein data expression analysis [9], which the expression of normal lung tissue and lung cancer were compared. 10 proteins (GFAP, STAT3, SRC, CD36, CAV1, PCNA, HDAC9, AQP1, APOA1, RALA) associated with dedifferentiation of Schwann cells were increased in lung cancer, including GFAP. No downregulated protein expression was associated with Schwann cell dedifferentiation.

### Cancer protein expression patterns correspond to pathway activation levels

We also performed an RPPA protein analysis. Only CDH1 and CDH2 protein expression data were available for analysis. We observed that CDH2 was overexpressed in LUSC compared to LUAD. Additionally, no significant difference was found in CDH1 analysis (Supplementary Figure 26).

In order to analyze the pathway by which Schwann cells induce neoplastic and their own cell proliferation and migration, we evaluated the expression of MEK1 (MEK1 and MEK1_pS217S221), ERK2, AKT (PRAS40_pT246, AKT_pT308 and AKT_pS473), RAF (CRAF and CRAF_pS338), and GSK3Β (GSK3_pS9 and GSK3ALPHABETA_pS21S9) proteins. We observed higher expression of ERK2, AKT, CRAF and GSK3Β proteins in LUSC when compared with LUAD. Only MEK1_pS217S221 presented higher expression in LUAD (Supplementary Figure 26).

### Prognostic analyses

**Figure 3:**
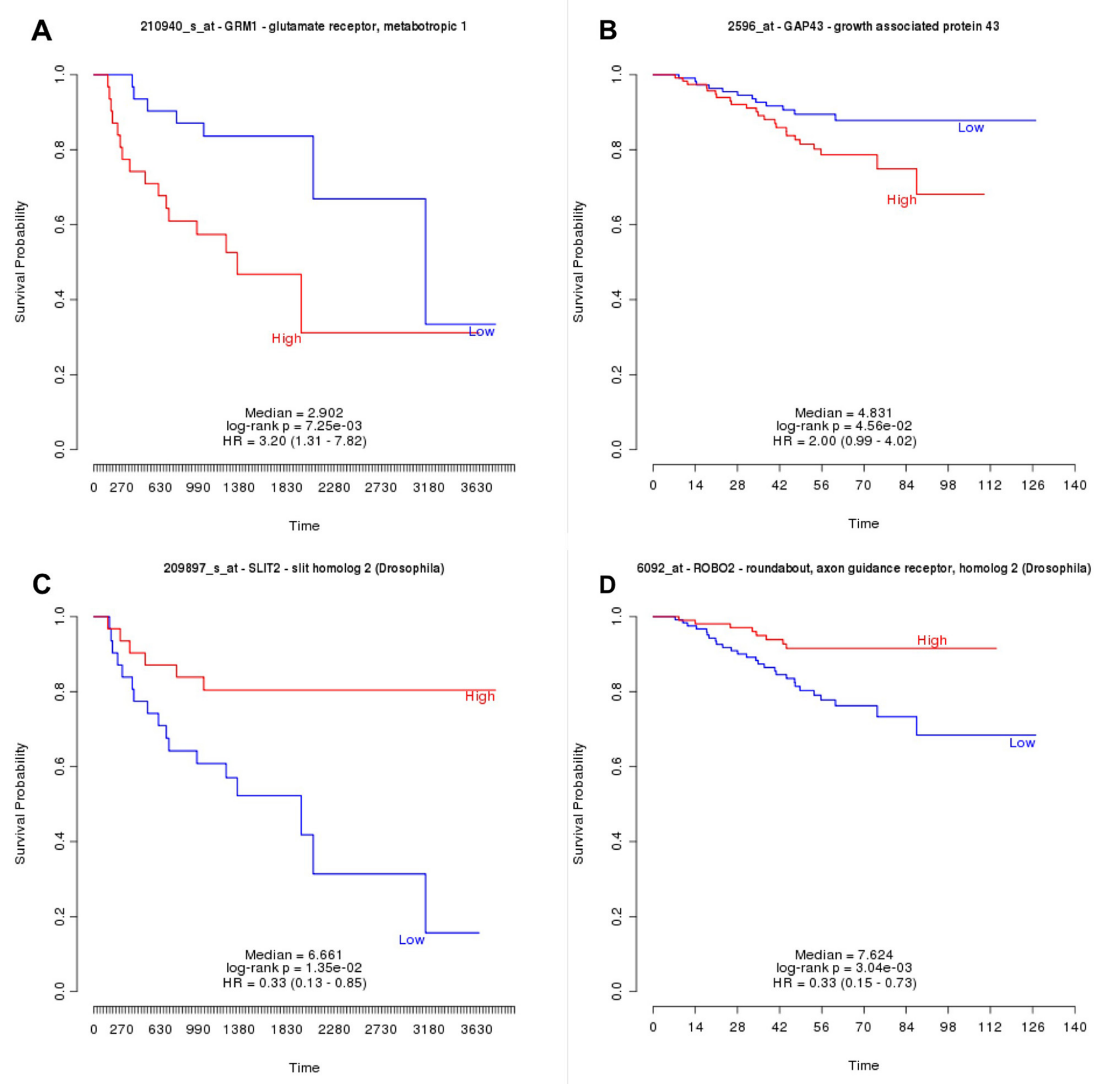
PRECOG Survival analyses in lung adenocarcinoma for Grm1. **(A)**, Gap43 **(B)**, Slit2 **(C)** and Robo2 **(D)** mRNA expression. Note that Grm1 and Gap43 upregulation and, Slit2 and Robo2 downregulation are associated with poor survival.

**Figure 4:**
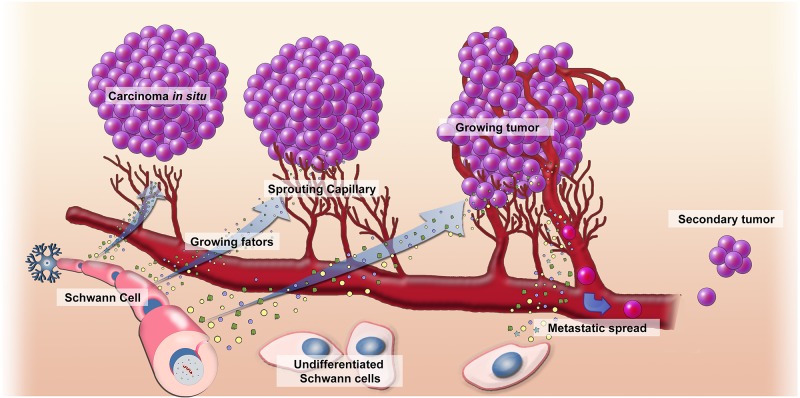
Mechanism of action of Schwann cells in the development of cancer. We suppose that Schwann cells assist the neoplastic lung cells to invade surrounding tissue during the early stages of carcinogenesis. This mechanism would be possible through the dedifferentiation of the Schwann cells and blocking axon guidance signaling pathways. Migration of Schwann cells to the peritumoral region would be associated with perineural invasion and, subsequently, central nervous system metastases.

TIMER survival analyses showed that *Ccne1*, *Mki67*, and *Gap43* mRNA higher expression are associated with poor LUAD prognostic (Supplementary Figure 27). Similarly, PRECOG analysis showed that *Grm1, Gap43* and* Mki67* higher mRNA expression is associated with poor prognostic in LUAD. However, PRECOG analysis demonstrated *Slit2* and *Robo2* downregulation are associated with poor survival in LUAD samples (Figure 3). Figure 4 illustrates the possible association between Schwann cells and lung cancer.

## DISCUSSION

Cells surrounding a tumor form a molecular microenvironment known as stroma. Stroma formation is influenced by tumor cells and, in turn, it influences tumor growth, migration, and invasion [17]. Depending on the characteristics of the primary tumor, the stroma, and the intrinsic ability of metastatic tumor cells to adapt to a new location, malignant cells use distinct mechanisms for proliferation, survival, and dissemination. These cells often reactivate the expression of genes employed during embryogenesis [17, 18]. In order to leave the primary tumor and disseminate to distant organs, metastatic cells lose the ability to adhere to adjacent cells, enhancing their migratory and invasive capacity. This mechanism is accompanied by several modifications in the expression of genes, such as loss of epithelial receptor expression and increased expression of mesenchymal markers, a phenomenon also known as epithelial-mesenchymal transition [18, 19].

We investigated the association between Schwann cells and those lung cancer types often associated with perineural invasion. Initially, we used the GEO DataSets platform from the GEO repository to identify a database reporting gene expression in Schwann cells in a neoplastic context. Briefly, the database contains the expression results from experiments in which two factors produced by tumor cells were added into cell cultures. Comparisons were made between samples from the first and third passages. We then used these data to perform differential gene expression analysis and crossed data from upregulated genes with differential expression data from LUAD and LUSC. After identifying the genes in common, we ran several analysis tools to identify molecular pathways associated to these genes. Interestingly, we noted that the “neuroactive ligand-receptor interaction” pathway was active in LUSC. Studies have demonstrated the association between neuroactive ligand-receptors in the control of Schwann cell differentiation as well as in neoplastic cell proliferation. For example, GRM1 can activate the RAS/MEK1/ERK/AKT/RAF/GSK3Β phosphorylation cascade [20, 21], leading to an increase in cell proliferation and invasion. *Grm1* was upregulated in LUSC, suggesting that this gene participates in lung cancer development.

In these context, it has also been demonstrated that neuroactive ligand-receptors are active during Wallerian degeneration of peripheral nerves. Together with macrophages, Schwann cells remove axon and myelin debris, and clear a path for subsequent axonal regrowth and nerve regeneration [22]. Tumor cells benefit from nerve regeneration machinery to promote cell proliferation, migration, and invasion. It has been reported that Schwann cells are induced to migrate to the region close to the tumor at the beginning of the carcinogenic process. It was also suggested that Schwann cells promote neoplastic invasion by direct contact with cancer cells, since paracrine signaling and matrix remodeling are not yet sufficient to induce the migration process [4, 23]. Cell-cell contact between Schwann cells and tumor tissue is necessary to potentiate the ability of neoplastic cells to penetrate into the underlying tissue [1, 3]. After contact, the degradation of the extracellular matrix by Schwann cells provokes the formation of tunnels or bands coated with laminin, due to Schwann cells’ capacity to express matrix metalloproteins, especially MMP2 and MMP9. The mechanism of extracellular matrix degradation that promotes neoplastic migration also depends on the production of NCAM1 and N-cadherin (CDH2) by Schwann cells [1, 7].

In the present study, we analyzed the expression of genes associated with the hallmark of cancer. The importance of analyzing these genes derives from the hypothesis that there may be a correlation between the presence of Schwann cells and the aggressiveness of the neoplastic cell. Increased expression of genes related to cell differentiation (*Cdh1* and *Cdh2*), motility (*Mmp9*), and proliferation (*Mki67*), mainly in LUSC, suggests this correlation. We observed that these genes are mutated and had higher expression in lung cancer samples when compared with normal tissue.

Decreased E-cadherin protein expression after contact of Schwann cells with the tumor resembles the mechanism followed by cells during axonal repair process [8]. The loss of E-cadherin during the epithelial-mesenchymal transition in cancer is associated with a positive regulation of NCAM1 and CDH2 [1, 3, 4, 8]. When E-cadherin is suppressed, NCAM1 and CDH2 are upregulated; they associate with the p59fyn protein, whose subsequent activation leads to inhibition of focal adhesion and an increase in cell migration. A study using oncogenic *K-ras* pancreatic cancer cell lines identified increased levels of polysialylated NCAM1 expression, which interacts with E-cadherin to create steric hindrance of homophilic binding and decrease cell adhesion [1]. In our study, we observed upregulation of E-cadherin and CDH2 in both lung cancers. A CDH2 protein RPPA analysis showed that it is overexpressed in LUSC. Copy number alteration analysis also showed that amplification and gain are associated with E-cadherin and N-cadherin mRNA levels in both cancers. However, we showed that *Cdh2* mRNA expression is higher in LUSC when compared to LUAD. No difference was observed neither in *Ncam1* mRNA nor in E-cadherin protein analyses.

Differentiation of myelinating Schwann cells may undergo interference from inhibitory pathways that negatively control the expression of genes responsible for myelin sheath formation. NOTCH1 and JUN stand out among the negative regulators of the myelination program, in the same way as SOX-2 and PAX-3 [2, 24, 25]. The myelinating phenotype also involves the inactivation of a number of genes linked to the production of immature Schwann cell markers. Some transcription factors are responsible for ensuring proper maturation in Schwann cells. SOX-10 acts synergistically with a second factor, OCT-6, resulting in the expression of *Krox-20*. In turn, KROX-20 is a key inducer of expression of myelin genes, such as *Mbp*, *Mpz* and *Prx*. The maintenance of the myelinating phenotype therefore requires the continuous expression of KROX-20 and SOX-10, considering that inactivation of both proteins results in dedifferentiation of Schwann cells [24].

Previous studies have shown that Schwann cells induce cellular aggressiveness in lung cancer [26]. During dedifferentiation, Schwann cells express proteins initially lost during the myelination process. Among these proteins are GAP43, NCAM1, P75NTR (*Ngfr*), GFAP and SOX-2 [2]. Data from our study showed that *Gfap*, *Ngfr* and *Gap43* gene expression is increased in lung cancers, especially in LUSC. To confirm our data, we performed an analysis by using protein expression data published before [9]. We selected only genes associated with Schwann cells dedifferentiation. The results showed that proteins associated with dedifferentiated Schwann cells are overexpressed in lung cancer, when compared with normal lung samples. Besides, *Gfap* and *Gap43* copy number alterations are associated with higher mRNA levels in LUAD and LUSC, respectively. It is likely that Schwann cells in these tissues are dedifferentiated, aiding tumors in their mechanisms of cell proliferation, migration, and tissue invasion.

During the process of carcinogenesis, there is an inactivation of the *Slit2* and *Robo2* genes, being therefore considered as tumor suppressor genes [27]. Both genes are extremely important during the process of nerve formation and repair. Both SLIT2 and ROBO2 have been reported to inhibit migration of Schwann cells [28]. In the present study, we observed that *Slit2* and *Robo2* mRNAs are decreased in lung cancer samples when compared to normal tissue. Also, there is a participation of miRNAs and of methylation of their promoter regions in the regulation of these genes. Similarly, both genes are targets for upregulated miRNAs. Immunohistochemical expression analyses revealed low expression of both genes in the lung. Thus, we suggest that decreased expression of *Slit2* and *Robo2* genes in lung cancer may favor the migration of Schwann cells; consequently, favoring invasion by neoplastic tissue.

We next analyzed the RAS/ MEK1/ ERK/ AKT/ RAF/ GSK3Β phosphorylation cascade. This pathway is activated during the proliferation process of Schwann cells and lung cancer. The authors observed that SC-treated lung cancer cells exhibit an increased phosphorylation of Akt and GSK-3β. Besides, they blocked CXCL5 expression in SC significantly decreases the phosphorylation of AKT and GSK-3β in SC-treated lung cancer cells; PI3K inhibitor LY294002 blocks the activation of AKT/GSK-3β signaling in SC-conditioned lung cancer cells. In our study, we observed that the RAS/ MEK1/ ERK/ AKT/ RAF/ GSK3Β phosphorylation is increased in LUSC when compared to LUAD, suggesting that, in LUSC samples, this pathway could be more active.

In summary, we observed that the “neuroactive ligand-receptor interaction” pathway was upregulated in lung squamous cell carcinoma and downregulated in lung adenocarcinoma. The “p53 signaling pathway” was active in both lung cancers, since *Ccne1*, *Cdkn2a*, and *Perp* were upregulated. Meanwhile, upregulated miRNAs inactivate the “axon guidance” pathway, targeting *Robo2* and *Slit2* genes. Both genes are also associated with Schwann cells migration inhibition. Also, *Gfap* and *Gap43* are overexpressed, leading to Schwann cells dedifferentiation. We believe that Schwann cells’ dedifferentiation and proliferation are induced by neoplastic tissue; consequently, Schwann cells produce different factors that will participate in several processes of tumor progression. These processes may also be involved in tumor invasion into the perineural tissue in lung cancer. Our data stimulate efforts in news studies to achieve the experimental and clinical validation about these biomolecules.

## MATERIALS AND METHODS

### Collection and inclusion criteria of studies

We searched the GEO database (https://www.ncbi.nlm.nih.gov/geo/) for publicly available studies. After a systematic review, only one GSE studies were retrieved. The inclusion criteria for studies were as follows: (1) experiments involving both cancer and Schwann cells and, (2) gene expression profiling of mRNA. Then, one gene expression profile (GSE4030) was collected for our analysis.

### Microarray data and Data processing

One gene expression profiles (GSE4030) was downloaded from the GEO database. GEO2R was applied to compare gene expression profiles in early and late passage human Schwann cells exposed to the cancer growth factors heregulin and forskolin. Because both groups in this comparison have been exposed to mitogens, differences in gene expression profiles will be interpreted as indicative of changes caused by prolonged versus short term exposure to mitogens.

GEO2R (http://www.ncbi.nlm.nih.gov/geo/geo2r/) is an interactive web tool for comparing two groups of data that can analyze any GEO series. The adjusted p-values using Benjamini and Hochberg false discovery rate method by default were applied to correct the occurrence of false positive results. An adj. P < 0.05 and a logFC ≥ 1 were set as the cut-off criteria.

### Gene list collection

We used Entrez Gene from NCBI (www.ncbi.nlm.nih.gov/gene/) and GeneCards (https://www.genecards.org/), which were used as the identifiers for Schwann cell dedifferentiation-related genes. The gene list and downregulated/ upregulated proteins of a previous published data [9] were combined and identified with a Venn Diagram 2.1.0 (http://bioinfogp.cnb.csic.es/tools/venny/index.html).

### Functional and pathway enrichment analysis

Gene ontology (GO) analysis of the relevant biological processes, cellular components, and molecular functions was performed using the protein analysis through evolutionary relationships program (PANTHER, www.pantherdb.org), a curated database of protein families, functions, and pathways. GO terms assigned into identified molecules were classified according to their functions [10].

The Kyoto Encyclopedia of Genes and Genomes (KEGG) is an integrated database resource for biological interpretation of genome sequences and other high-throughput data [11]. KEGG analyses were available at the DAVID database (https://david.ncifcrf.gov/), a data resource composed of an integrated biology knowledge base and analysis tools to extract meaningful biological information from large quantities of genes and protein collections. A *p*-value < 0.05 was set as the cut-off criterion [12].

### RNA-seq and clinical information data from The Cancer Genome Atlas (TCGA)

We used TCGAbiolinks, an R/Bioconductor software (http://bioconductor.org/packages/release/bioc/ html/TCGAbiolinks.html) [13] and the interphase TCGAbiolinksGUI [14] to download genomic and clinical data of both normal and solid tumor tissues for two different types of cancer from TCGA. Selected cancer types were lung squamous cell carcinoma (LUSC) and lung adenocarcinoma (LUAD). We retrieved level data for raw count mRNA and miRNA expression (Illumina HiSeq 2000). Co-expressed upregulated and downregulated DEGs from the gene expression profiles were combined and identified with a Venn Diagram 2.1.0 (http://bioinfogp.cnb.csic.es/tools/venny/index.html). An adj. P < 0.05 and a logFC ≥ 1 were set as the cut-off criteria.

DNA methylation analyses were performed using MEXPRESS dataset (https://mexpress.be/?ref=labworm). The cancer dataset, consisting of DNA methylation data (Illumina Infinium Human Methylation 450 K Bead array, Illumina, USA) and the β value, was considered as significantly hypermethylated only if the value was found in more than 5% of the tumors [15]. Copy number alteration analysis was performed using the cBio Cancer Genomics Portal (http://cbioportal.org).

### Prediction of miRNA targets

The target miRNAs of the DEGs from TCGA data were predicted with miRDB (http://mirdb.org/miRDB/), which is an online database for predicting microRNA targets [16].

### Lung cancer expression analyses

To obtain individual gene-protein data, relevant information from The Cancer Proteome Atlas (TCPA) website (https://tcpaportal.org/tcpa/analysis.html) was used as the primary source of information for reverse phase protein array (RPPA) analysis. Several other web resources were used as source of information while some more were used as analysis tools for lung cancer expression studies: Immunohistochemistry image-based protein data for both normal and cancer samples are available at the Human Protein Atlas (https://www.proteinatlas.org/). LOCATE database for protein subcellular location was included on the analysis (http://locate.imb.uq.edu.au/cgi-bin/sort_search.cgi). Survival analysis of the TCGA data was performed using the Survival module of the Tumor Immune Estimation Resource (TIMER) and Prediction of clinical outcomes from genomics (PRECOG). Kaplan–Meier plots were drawn using TIMER to explore the association between clinical outcome and gene expression, and to visualize survival differences.

### Statistical analysis

Statistical analyses involving copy number variation were performed using GraphPad Prism 7 software. A two-way ANOVA and a two-tailed unpaired *t* test were used to compare the means between groups.

## SUPPLEMENTARY MATERIALS FIGURES AND TABLES


