# Supplementary material for: Schwann cell reprogramming and lung cancer progression: a meta-analysis of transcriptome data
Source: Oncotarget. 2019 Dec 31;10(68):7288–307. doi: 10.18632/oncotarget.27204 (PMC6944448; doi:10.18632/oncotarget.27204)
Supplement: Supplementary file 1 [file oncotarget-10-7288-s001.pdf]

Schwann cell reprogramming and lung cancer progression: a meta-analysis of transcriptome data

SUPPLEMENTARY MATERIALS

Supplementary Table 1: Functional annotation analysis of downregulated differentially expressed genes (DEGs) in Schwann cells datasets using the DAVID tool

Term	Count	P value	Genes	Pop Hits	Fold Enrichment	Bonferroni
hsa05200:Pathways in cancer	31	6,88E-04	WNT5A, FGFR1, FGF18, FGF7, WNT5B, PGF, FGF11, BCL2L1, TGFB1, TCF7L1, CTNNB1, IGF1R, BCL2, HHIP, WNT8B, FN1, EGFR, TGFBR1, CBL, RUNX1T1, LEF1, BIRC5, FZD5, STK4, CDK2, PRKCB, PLCG2, PDGFRA, JAK1, WNT9A, ITGA2B	328	1,899583052	0,096222132
hsa04510:Focal adhesion	22	8,54E-04	EGFR, FLT1, PGF, COL3A1, ITGB4, ITGA11, ITGB5, ACTN2, CTNNB1, PRKCB, IGF1R, CCND3, CCND2, RASGRF1, COMP, BCL2, COL6A3, PDGFRA, RELN, COL1A1, FN1, ITGA2B	201	2,199870214	0,118016284
hsa05412: Arrhythmogenic right ventricular cardiomyopathy (ARVC)	11	0,0039537	SLC8A1, SGCG, ITGB4, ITGA11, LEF1, ITGB5, ACTN2, CACNA1C, TCF7L1, ITGA2B, CTNNB1	76	2,909038902	0,441413264
hsa04520:Adherens junction	11	0,00435076	EGFR, IGF1R, FGFR1, TGFBR1, LMO7, LEF1, ACTN2, INSR, TCF7L1, FARP2, CTNNB1	77	2,871259176	0,473211952
hsa05217:Basal cell carcinoma	9	0,00522849	WNT5A, WNT5B, LEF1, WNT9A, HHIP, FZD5, TCF7L1, CTNNB1, WNT8B	55	3,288896874	0,537266618
hsa04115:p53 signaling pathway	10	0,00591844	CCNB2, CCND3, CCND2, RRM2, ATR, GADD45B, IGFBP3, GTSE1, CDK2, TP53AIP1	68	2,955707975	0,582134333
hsa05210:Colorectal cancer	11	0,00808527	EGFR, IGF1R, BCL2, TGFBR1, PDGFRA, LEF1, BIRC5, FZD5, TCF7L1, TGFB1, CTNNB1	84	2,631987578	0,696801067
hsa04914:Progesterone-mediated oocyte maturation	11	0,00950996	PGR, IGF1R, ADCY4, RPS6KA6, CCNB2, ANAPC5, ADCY8, BUB1, PDE3A, CDC25C, CDK2	86	2,570778564	0,754548123

(*Continued*)

Term	Count	P value	Genes	Pop Hits	Fold Enrichment	Bonferroni
hsa04114:Oocyte meiosis	12	0,01958301	PGR, IGF1R, ADCY4, RPS6KA6, CCNB2, ANAPC5, ADCY8, BUB1, FBXO5, PTTG2, CDC25C, CDK2	110	2,192597916	0,945374938
hsa04340:Hedgehog signaling pathway	8	0,01961551	WNT5A, WNT5B, WNT9A, HHIP, LRP2, BMP7, IHH, WNT8B	56	2,871259176	0,945640478
hsa04512:ECM-receptor interaction	10	0,02228893	COMP, COL6A3, COL3A1, ITGB4, ITGA11, ITGB5, RELN, COL1A1, ITGA2B, FN1	84	2,39271598	0,963612841
hsa04916: Melanogenesis	11	0,02390153	WNT5A, ADCY4, WNT5B, ADCY8, LEF1, WNT9A, FZD5, TCF7L1, PRKCB, CTNNB1, WNT8B	99	2,233201581	0,971452794
hsa04020:Calcium signaling pathway	16	0,0271791	EGFR, PTGER1, ADCY4, SLC8A1, NOS1, ADCY8, CACNA1I, PTGFR, PRKCB, EDNRA, AGTR1, ATP2A3, RYR3, PLCG2, PDGFRA, CACNA1C	176	1,82716493	0,982588545
hsa05414:Dilated cardiomyopathy	10	0,03744429	ADCY4, SLC8A1, SGCG, ADCY8, ITGB4, ITGA11, ITGB5, CACNA1C, TGFB1, ITGA2B	92	2,184653721	0,996338966
hsa04110:Cell cycle	12	0,04460437	CDC45, CCNB2, CCND3, ANAPC5, CCND2, BUB1, PTTG2, ATR, CDC25C, GADD45B, CDK2, TGFB1	125	1,929486166	0,998778376
hsa05014:Amyotrophic lateral sclerosis (ALS)	7	0,04558666	ALS2, SLC1A2, NOS1, GRIA2, BCL2, RAB5A, BCL2L1	53	2,65456037	0,998949816

The results revealed that downregulated consistent DEGs indicated the genes that were mainly associated with “Pathways in cancer” and, “Focal adhesion”.

Supplementary Table 2: Functional annotation analysis of upregulated differentially expressed genes (DEGs) in Schwann cells datasets using the DAVID tool

Term	Count	P value	Genes	Pop Hits	Fold Enrichment
hsa04080:Neuroactive ligand-receptor interaction	24	1,95E-06	CSH1, MCHR1, F2RL3, GABRG3, PTH2R, GRIK1, GABRA4, GABRB2, GLRA3, GABRA5, BRS3, FPR3, GRM1, TAAR8, GH2, GH1, SSTR5, APLNR, P2RY10, CHRM3, GRIA2, CHRM1, ADRA1A, GPR50	256	3,075604839
hsa04950:Maturity onset diabetes of the young	4	0,03846734	HES1, HHEX, HNF1A, FOXA2	25	5,249032258
hsa00350:Tyrosine metabolism	5	0,04300193	DDC, PNMT, ADH1B, DBH, FAH	44	3,728005865

The results revealed that upregulated consistent DEGs indicated the genes that were associated with “Neuroactive ligand-receptor interaction”, “Maturity onset diabetes of the young” and, “Tyrosine metabolism”.

Supplementary Table 3: Functional annotation analysis of downregulated differentially expressed (DEGs) overlapped genes in Schwann cells and lung adenocarcinoma datasets using the DAVID tool

Term	Count	P value	Genes	Pop Hits	Fold Enrichment
hsa04080:Neuroactive ligand-receptor interaction	24	1,95E-06	CSH1, MCHR1, F2RL3, GABRG3, PTH2R, GRIK1, GABRA4, GABRB2, GLRA3, GABRA5, BRS3, FPR3, GRM1, TAAR8, GH2, GH1, SSTR5, APLNR, P2RY10, CHRM3, GRIA2, CHRM1, ADRA1A, GPR50	256	3,07560484
hsa04950:Maturity onset diabetes of the young	4	0,03846734	HES1, HHEX, HNF1A, FOXA2	25	5,24903226
hsa00350:Tyrosine metabolism	5	0,04300193	DDC, PNMT, ADH1B, DBH, FAH	44	3,72800587
hsa00740:Riboflavin metabolism	3	0,08287513	ACP5, MTMR7, ACPP	16	6,15120968
hsa04020:Calcium signaling pathway	10	0,08489932	ATP2B2, PLCB4, CHRM3, CHRM1, ADRA1A, GNAS, CACNA1E, CAMK2B, CACNA1C, GRM1	176	1,86400293

The results revealed that downregulated consistent DEGs indicated the genes that were associated with "Neuroactive ligand-receptor interaction", "Maturity onset diabetes of the young" and, "Tyrosine metabolism".

Supplementary Table 4: Functional annotation analysis of upregulated differentially expressed (DEGs) overlapped genes in Schwann cells and lung adenocarcinoma datasets using the DAVID tool

Term	Count	P value	Genes	Pop Hits	Fold Enrichment
hsa04115:p53 signaling pathway	3	0,02105574	CCNE1, CDKN2A, PERP	68	12,4632353
hsa00340:Histidine metabolism	2	0,09279159	DDC, ACY3	29	19,4827586

No pathways were associated with upregulated DEGs.

Supplementary Table 5: Functional annotation analysis of downregulated differentially expressed (DEGs) overlapped genes in Schwann cells and Lung squamous cell carcinoma datasets using the DAVID tool

Term	Count	P value	Genes	Pop Hits	Fold Enrichment
hsa04270:Vascular smooth muscle contraction	4	0,05024756	AGTR1, ADCY4, MYH11, PPP1R14A	112	4,65659341
hsa04340:Hedgehog signaling pathway	3	0,06506959	WNT9A, HHIP, IHH	56	6,98489011

No pathways were associated with upregulated DEGs.

Supplementary Table 6: Functional annotation analysis of upregulated differentially expressed (DEGs) overlapped genes in Schwann cells and Lung squamous cell carcinoma datasets using the DAVID tool

Term	Count	P value	Genes	Pop Hits	Fold Enrichment
hsa04080:Neuroactive ligand-receptor interaction	5	0,02607792	PTH2R, CHRM3, GABRA5, GPR50, GRM1	256	4,13818359
hsa04115:p53 signaling pathway	3	0,03721193	CCNE1, CDKN2A, PERP	68	9,34742647

The results revealed that upregulated consistent DEGs indicated the genes that were associated with "Neuroactive ligand-receptor interaction" and, "p53 signaling pathway".

Supplementary Table 7: Functional annotation analysis of downregulated miRNA target genes combined with upregulated genes in lung adenocarcinoma datasets using the DAVID tool

Term	Count	P value	Genes	Pop Hits	Fold Enrichment
hsa04360:Axon guidance	8	8,85E-04	SEMA5A, DCC, SEMA6A, SEMA6D, CFL2, ROBO2, CXCL12, SLIT2	129	5,0055371
hsa04010:MAPK signaling pathway	10	0,00463642	DUSP1, FGF14, JUN, PTPN5, MRAS, NTRK2, TGFBR2, RRAS, CACNB4, DUSP8	267	3,02300696
hsa04520:Adherens junction	4	0,06686838	PTPRM, WASF3, TGFBR2, LMO7	77	4,19294991

The results revealed that downregulated target genes indicated the genes that were associated with "Axon guidance" and, "MAPK signaling pathway".

Supplementary Table 8: Functional annotation analysis of upregulated miRNA target genes combined with downregulated genes in lung adenocarcinoma datasets using the DAVID tool

Term	Count	P value	Genes	Pop Hits	Fold Enrichment
hsa04080:Neuroactive ligand-receptor interaction	9	0,00512739	GPR83, CALCR, MCHR1, GABRR1, SSTR3, PRLR, PTH2R, GRIK3, GRIN2A	256	3,25035511
hsa05200:Pathways in cancer	9	0,0211608	E2F1, E2F2, CKS1B, RET, HSP90B1, SLC2A1, SKP2, FGF11, WNT8B	328	2,53686253
hsa05222:Small cell lung cancer	4	0,05907025	E2F1, E2F2, CKS1B, SKP2	84	4,4025974

The results revealed that upregulated target genes indicated the genes that were associated with "Neuroactive ligand-receptor interaction" and, "Pathways in cancer".

Supplementary Table 9: Functional annotation analysis of downregulated miRNA target genes combined with upregulated genes in Lung squamous cell carcinoma datasets using the DAVID tool

Term	Count	P value	Genes	Pop Hits	Fold Enrichment
hsa05414:Dilated cardiomyopathy	12	0,00134872	ITGA9, ADRB1, ADCY9, ITGA1, RYR2, SGCD, CACNB4, TTN, CACNA1C, CACNA1D, CACNA2D2, TGFB2	92	3,12858901
hsa04270:Vascular smooth muscle contraction	13	0,00215565	ADORA2A, PRKG1, PRKCE, ITPR1, PRKCB, PRKCQ, GNAQ, ADCY9, GUCY1A2, CALCRL, CACNA1C, PLCB2, CACNA1D	112	2,78407177
hsa05410:Hypertrophic cardiomyopathy (HCM)	11	0,00250592	ITGA9, IL6, ITGA1, RYR2, SGCD, CACNB4, TTN, CACNA1C, CACNA1D, CACNA2D2, TGFB2	85	3,10405105
hsa04020:Calcium signaling pathway	16	0,00599574	GNA14, ERBB4, BST1, ADORA2A, ITPR1, PRKCB, EDNRB, PLCE1, ADRB2, ADRB1, GNAQ, ADCY9, RYR2, CACNA1C, PLCB2, CACNA1D	176	2,18053173
hsa04530:Tight junction	13	0,00924044	PARD6B, CLDN18, MAGI3, MAGI1, MPDZ, PRKCE, PRKCB, EPB41L2, PRKCQ, EPB41L3, TJP1, JAM2, MYH10	134	2,32698536
hsa04540:Gap junction	10	0,01095339	TJP1, ADRB1, GNAQ, ADCY9, GUCY1A2, PRKG2, PRKG1, PLCB2, ITPR1, PRKCB	89	2,69503922
hsa04060:Cytokine-cytokine receptor interaction	20	0,01177253	IL18R1, IL6, BMP2, CXCL5, IL6ST, FLT4, LEPR, CSF1, TGFBR2, BMPR2, LIFR, IL6R, HGF, CXCL12, TGFB2, KDR, CCL13, TNFRSF10D, PLEKHO2, CSF2RA	262	1,83098084
hsa04010:MAPK signaling pathway	20	0,01419803	MEF2C, FGF7, FGF14, TGFBR2, CACNB4, CACNA2D2, TGFB2, PRKCB, FOS, BDNF, DUSP1, RASGRP3, RPS6KA2, JUN, MAP3K8, CACNA1C, NFATC2, RAPGEF2, CACNA1D, FGF2	267	1,79669281
hsa04360:Axon guidance	12	0,01751762	SEMA5A, PLXNC1, NRP1, SEMA3G, ABLIM3, CFL2, SEMA3E, ROBO2, UNC5C, NFATC2, CXCL12, SLIT2	129	2,23124177
hsa04514:Cell adhesion molecules (CAMs)	12	0,02046188	NCAM2, ITGA9, CLDN18, PTPRM, CADM1, CD34, HLA-DPA1, JAM2, SDC4, NEGR1, HLA-DQA2, HLA-DQA1	132	2,18053173
hsa04730:Long-term depression	8	0,02335132	GNAQ, GRIA1, GUCY1A2, PRKG2, PRKG1, PLCB2, ITPR1, PRKCB	69	2,78096801
hsa04640:Hematopoietic cell lineage	9	0,02540041	CD55, IL6, CD34, CSF1, FCGR1A, ITGA1, IL6R, CSF2RA, CD1E	86	2,51014699
hsa04630:Jak-STAT signaling pathway	13	0,02663018	PIK3CG, SPRY1, IL6, SOCS2, SOCS3, IL6ST, STAT5A, LEPR, LIFR, IL6R, CSF2RA, SPRY4, CISH	155	2,01171637
hsa05200:Pathways in cancer	22	0,02976063	PIK3CG, IL6, BMP2, FGF7, FGF14, STAT5A, MITF, TGFBR2, RUNX1T1, HGF, FZD5, FZD4, TGFB2, DAPK1, PRKCB, FOS, ETS1, JUN, RARA, AXIN2, FGF2, CSF2RA	328	1,60880695
hsa05412:Arrhythmogenic right ventricular cardiomyopathy (ARVC)	8	0,03717375	ITGA9, ITGA1, RYR2, SGCD, CACNB4, CACNA1C, CACNA1D, CACNA2D2	76	2,52482622
hsa04930:Type II diabetes mellitus	6	0,04376766	PIK3CG, SOCS2, SOCS3, CACNA1C, PRKCE, CACNA1D	47	3,06202328
hsa04912:GnRH signaling pathway	9	0,04959421	GNAQ, ADCY9, JUN, HBEGF, CACNA1C, PLCB2, CACNA1D, ITPR1, PRKCB	98	2,20278206

The results revealed that downregulated target genes indicated the genes that were associated with “Dilated cardiomyopathy” and, “Vascular smooth muscle contraction”.

Supplementary Table 10: Functional annotation analysis of upregulated miRNA target genes combined with downregulated genes in Lung squamous cell carcinoma datasets using the DAVID tool

Term	Count	P value	Genes	Pop Hits	Fold Enrichment
hsa00030:Pentose phosphate pathway	5	8,37E-04	PGM2, GPI, G6PD, PGD, PRPS2	25	11,3
hsa04110:Cell cycle	7	0,02160488	E2F2, YWHAZ, YWHAQ, SKP2, CDK6, MCM4, CDK2	125	3,164
hsa05200:Pathways in cancer	12	0,02689791	EGFR, BID, WNT5A, E2F2, CKS1B, WNT7B, SKP2, FGF11, TFG, LEF1, CDK6, CDK2	328	2,06707317
hsa04310:Wnt signaling pathway	7	0,04798391	WNT5A, TBL1XR1, WNT7B, DKK1, SFRP1, SFRP2, LEF1	151	2,6192053
hsa05222:Small cell lung cancer	5	0,05876079	E2F2, CKS1B, SKP2, CDK6, CDK2	84	3,36309524

The results revealed that upregulated target genes indicated the genes that were associated with "Pentose phosphate pathway", "Cell cycle", "Pathways in cancer", and "Wnt signaling pathway".

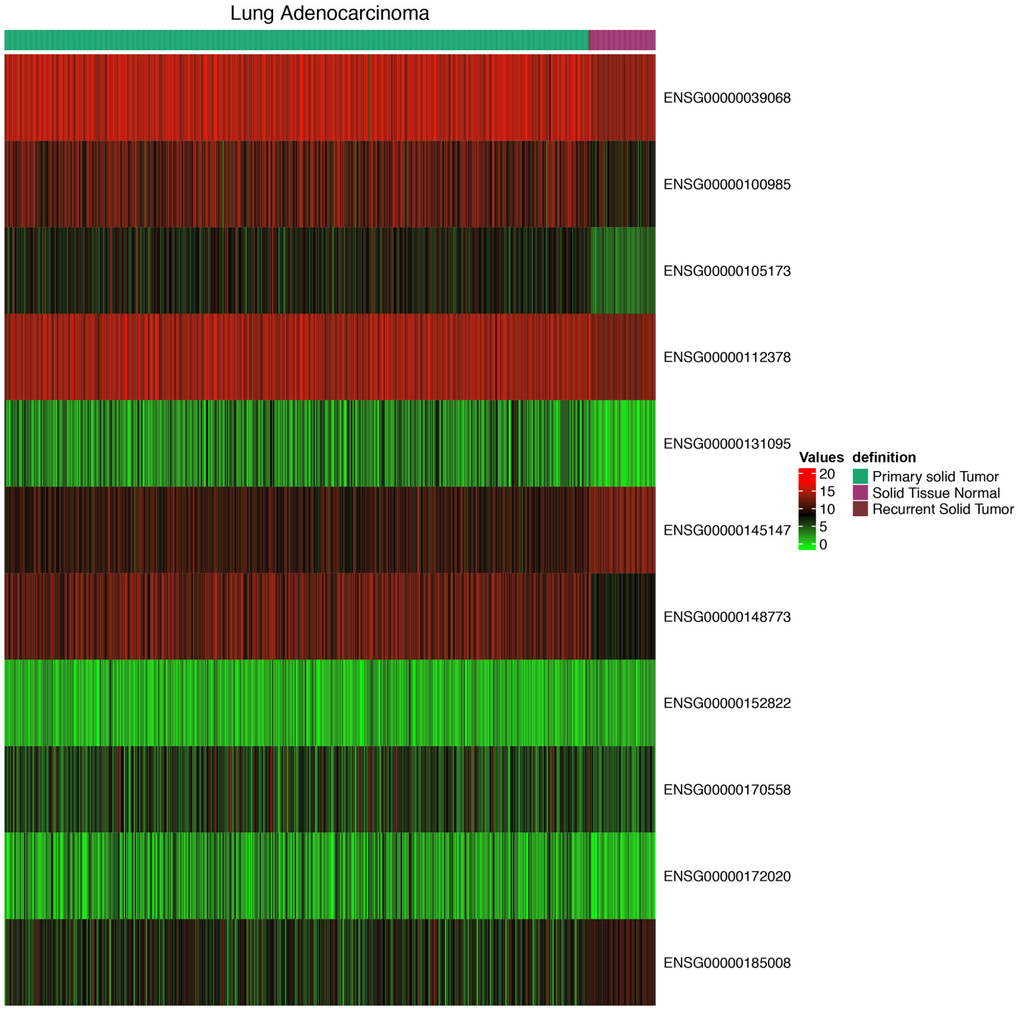

Supplementary Figure 1: Heatmap of *Cdh1*, *Mmp9*, *Ccne1*, *Perp*, *Gfap*, *Slit2*, *Mki67*, *Grm1*, *Cdh2*, *Gap43* and, *Robo2* differentiated expressed genes (DEGs) in LUSC samples.

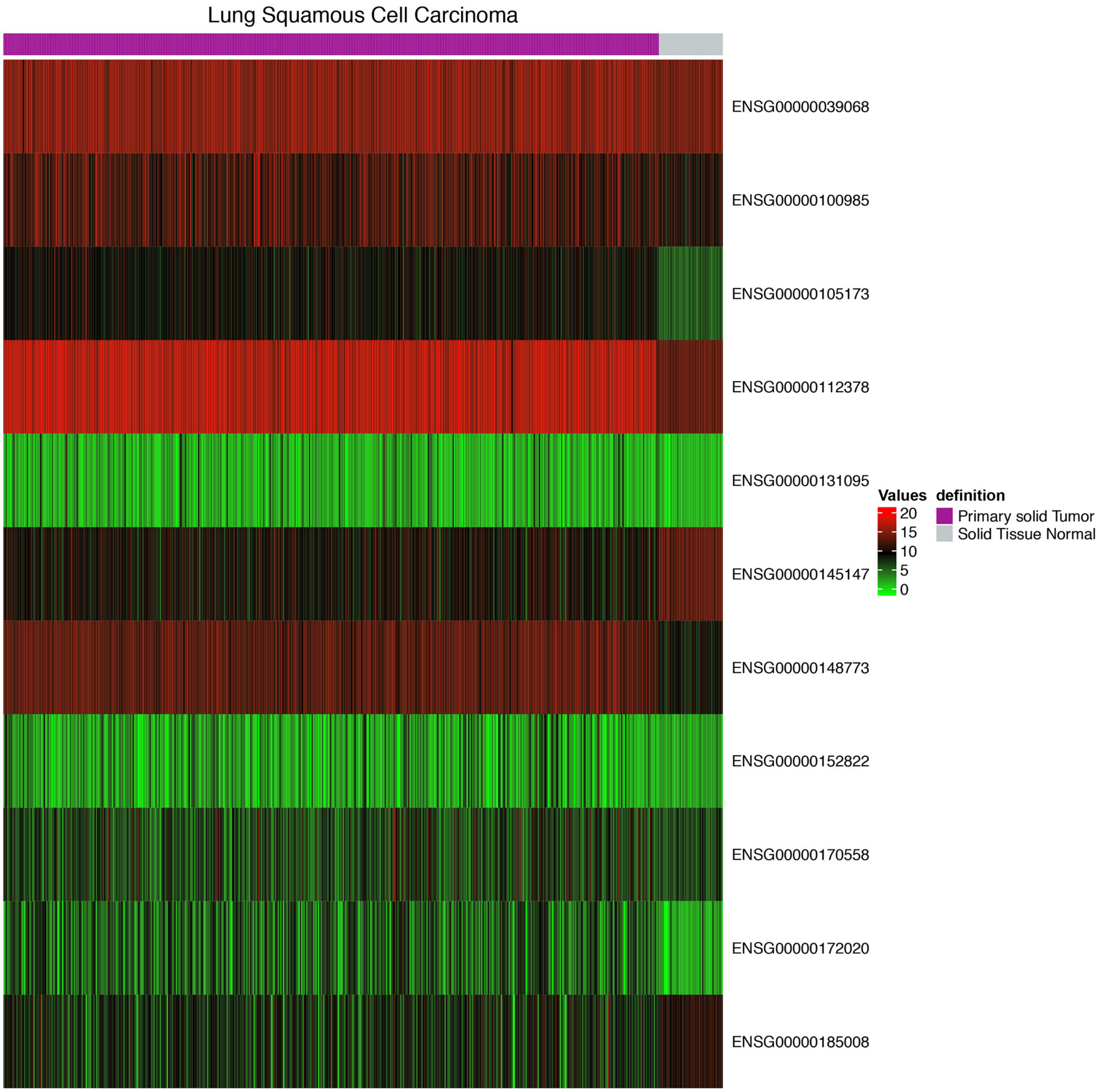

Supplementary Figure 2: Heatmap of *Cdh1*, *Mmp9*, *Ccne1*, *Perp*, *Gfap*, *Slit2*, *Mki67*, *Grm1*, *Cdh2*, *Gap43* and, *Robo2* differentiated expressed genes (DEGs) in in LUAD samples.

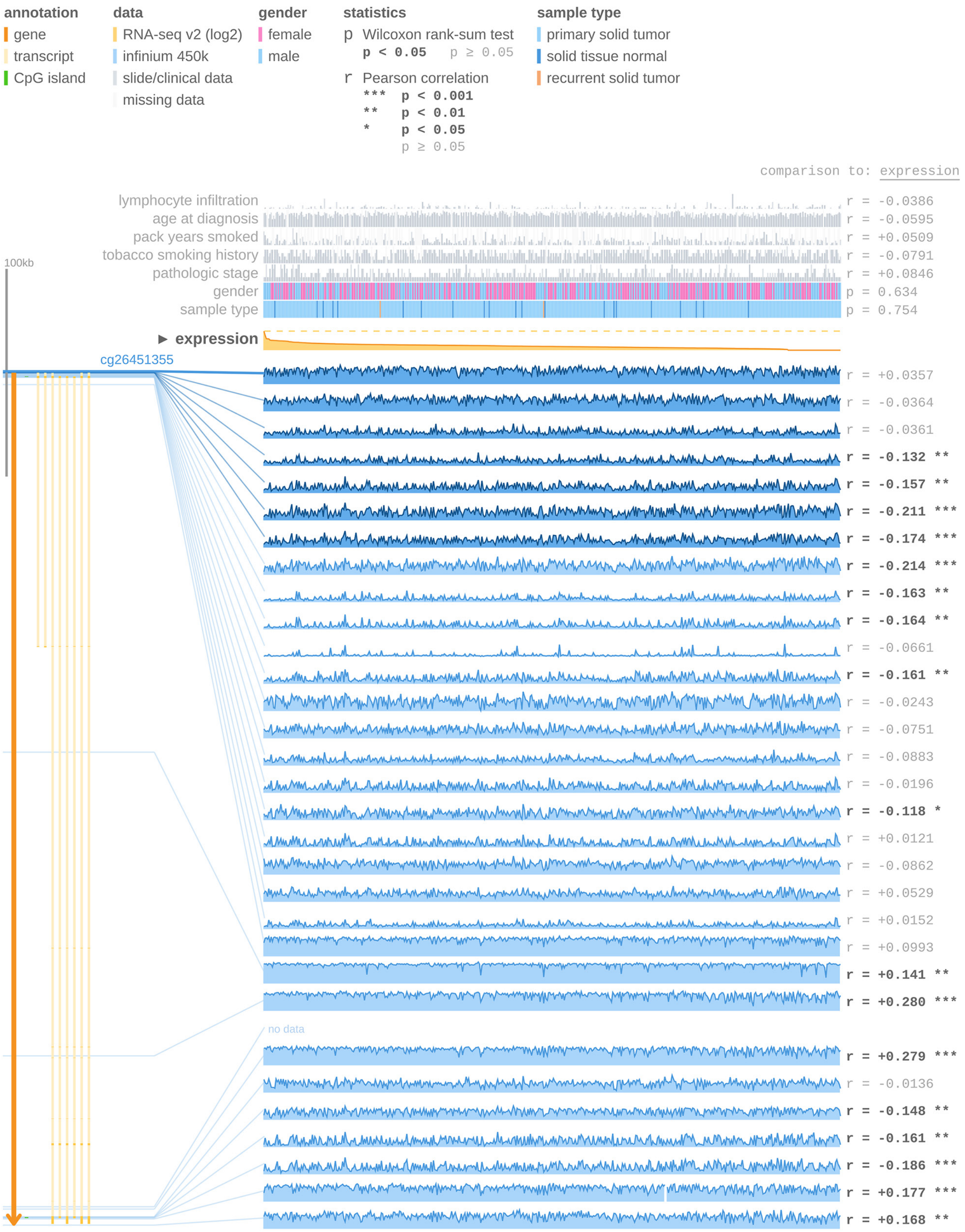

Supplementary Figure 3: Methylation analysis of *Grm1* gene in lung adenocarcinoma. The highlighted probes are promoter gene region.

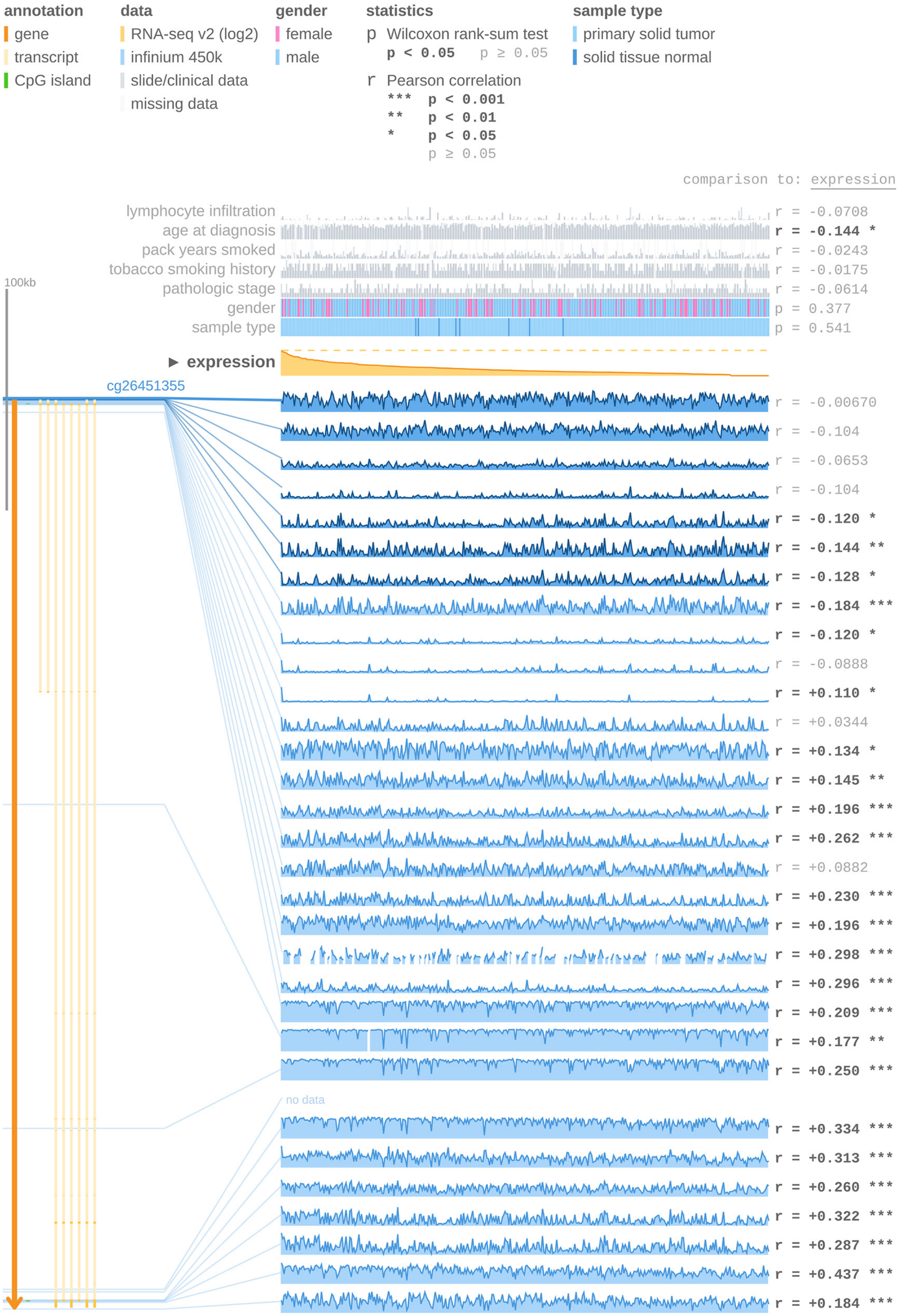

Supplementary Figure 4: Methylation analysis of *Grm1* gene in Lung squamous cell carcinoma. The highlighted probes are promoter gene region.

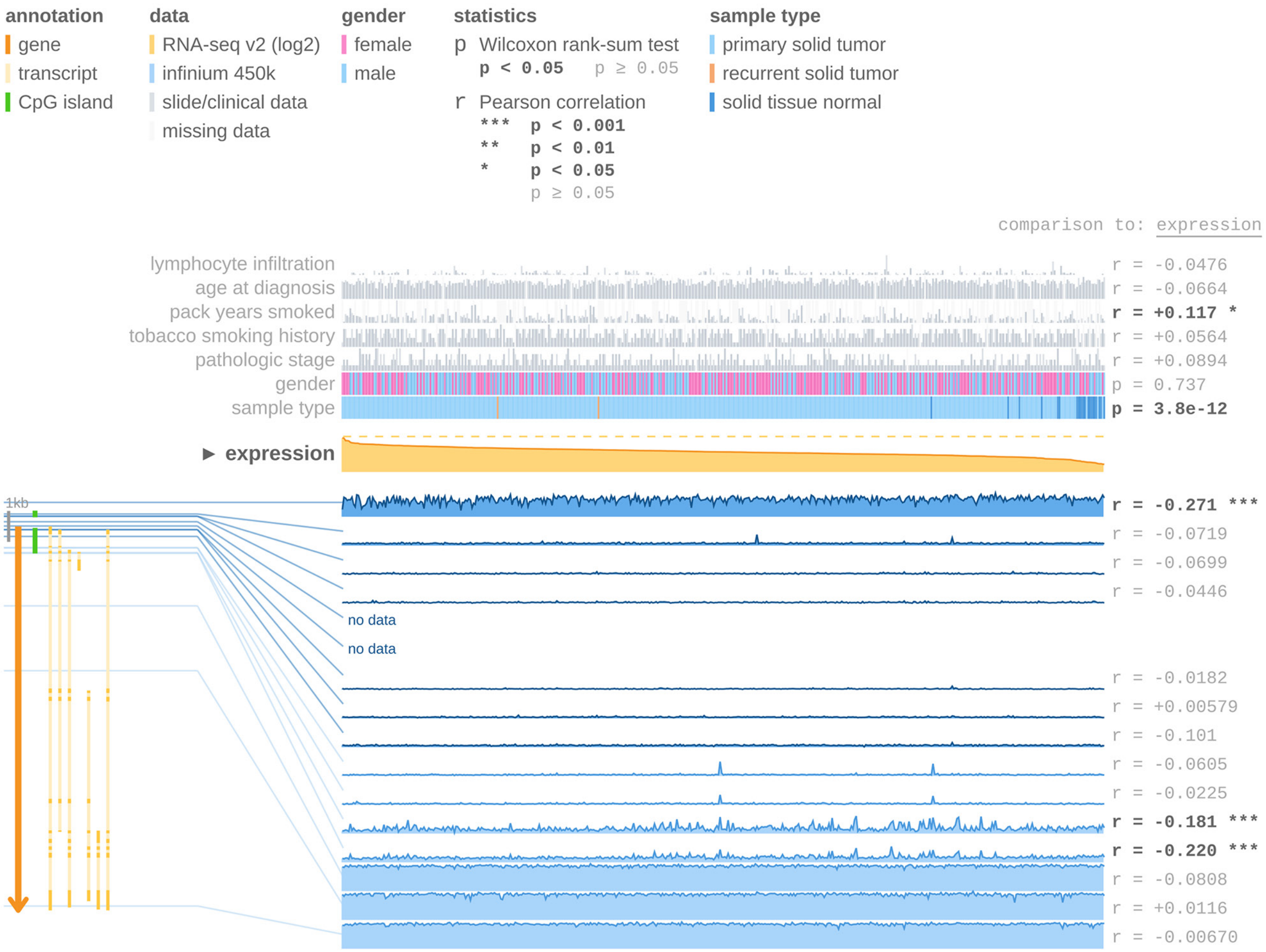

Supplementary Figure 5: Methylation analysis of *Ccne1* gene in lung adenocarcinoma. The highlighted probes are promoter gene region.

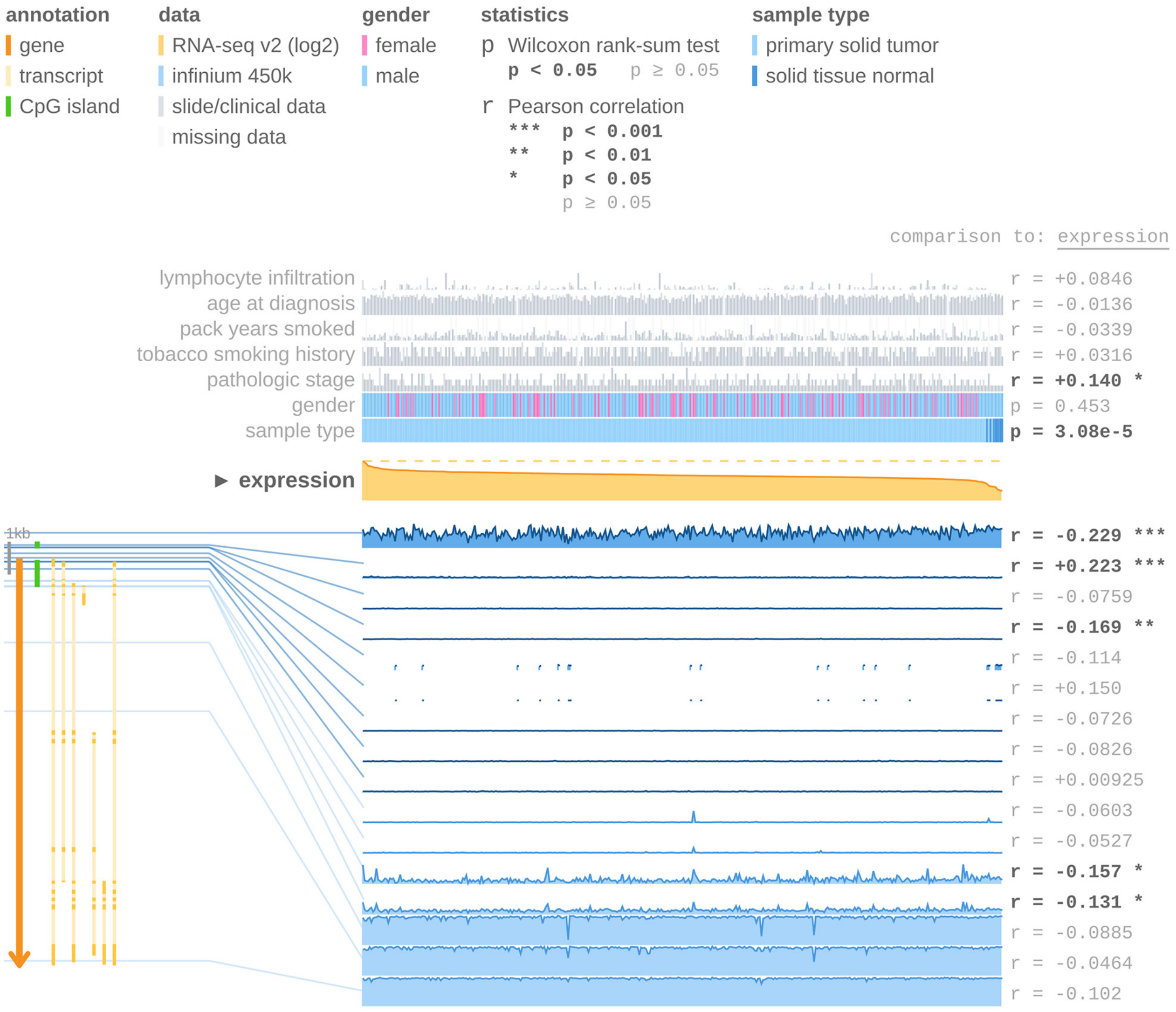

Supplementary Figure 6: Methylation analysis of *Ccne1* gene in Lung squamous cell carcinoma. The highlighted probes are promoter gene region.

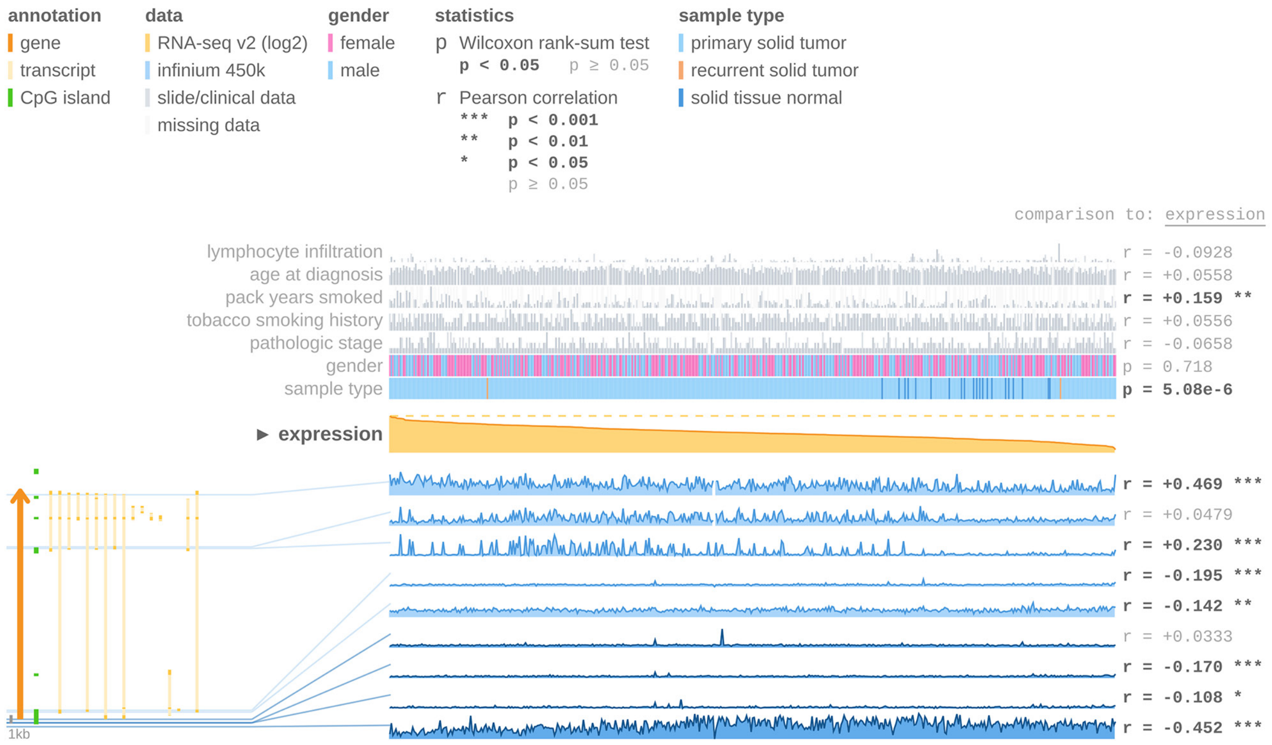

Supplementary Figure 7: Methylation analysis of *Cdkn2a* gene in lung adenocarcinoma. The highlighted probes are promoter gene region.

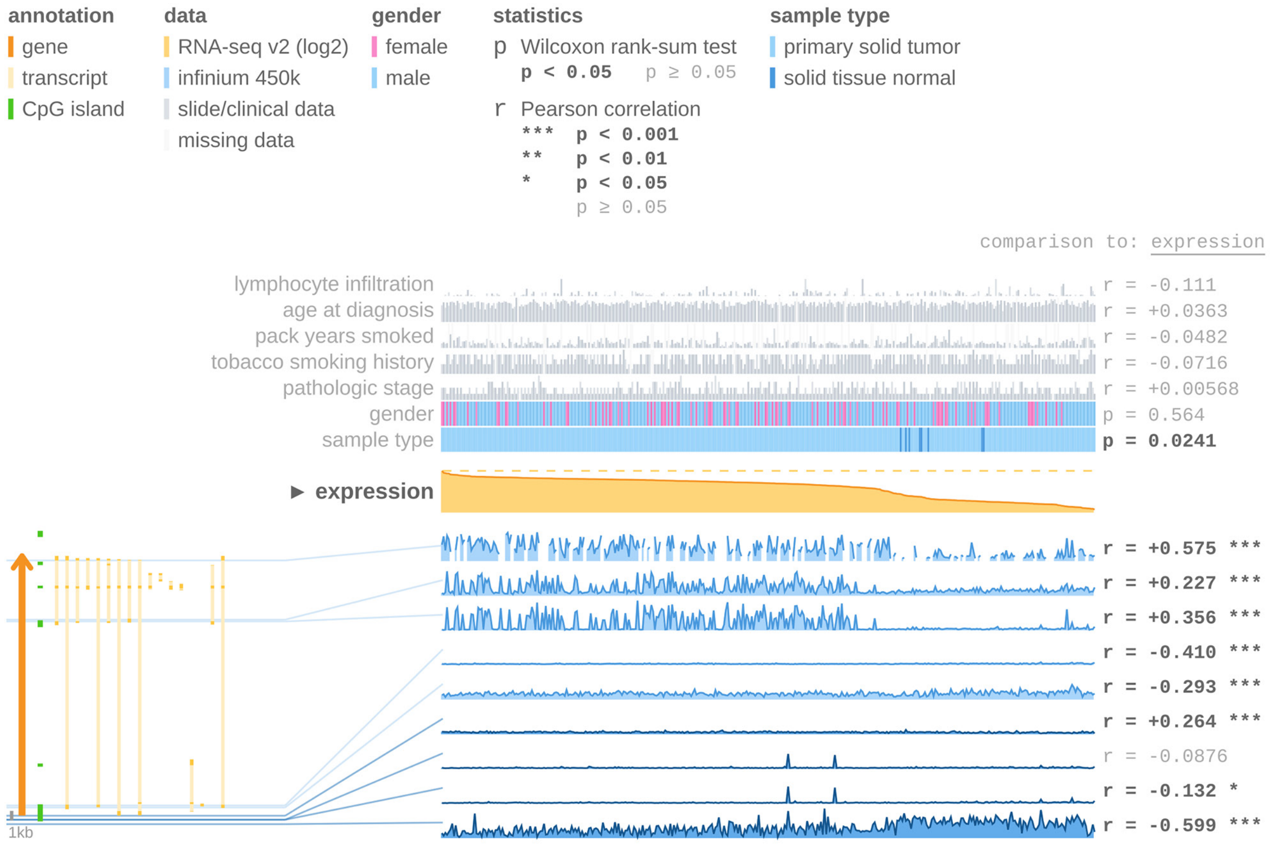

Supplementary Figure 8: Methylation analysis of *Cdkn2a* gene in Lung squamous cell carcinoma. The highlighted probes are promoter gene region.

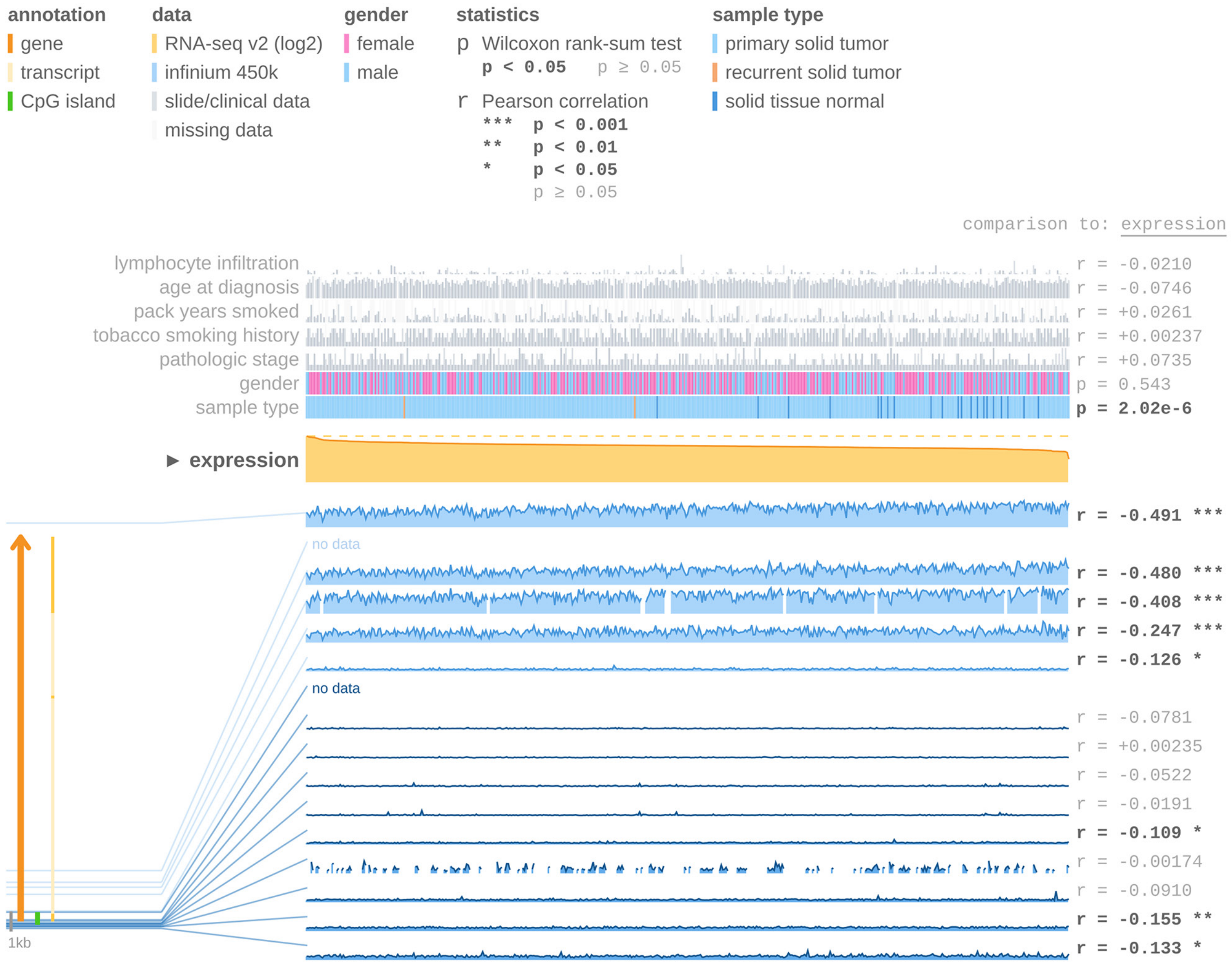

Supplementary Figure 9: Methylation analysis of *Perp* gene in lung adenocarcinoma. The highlighted probes are promoter gene region.

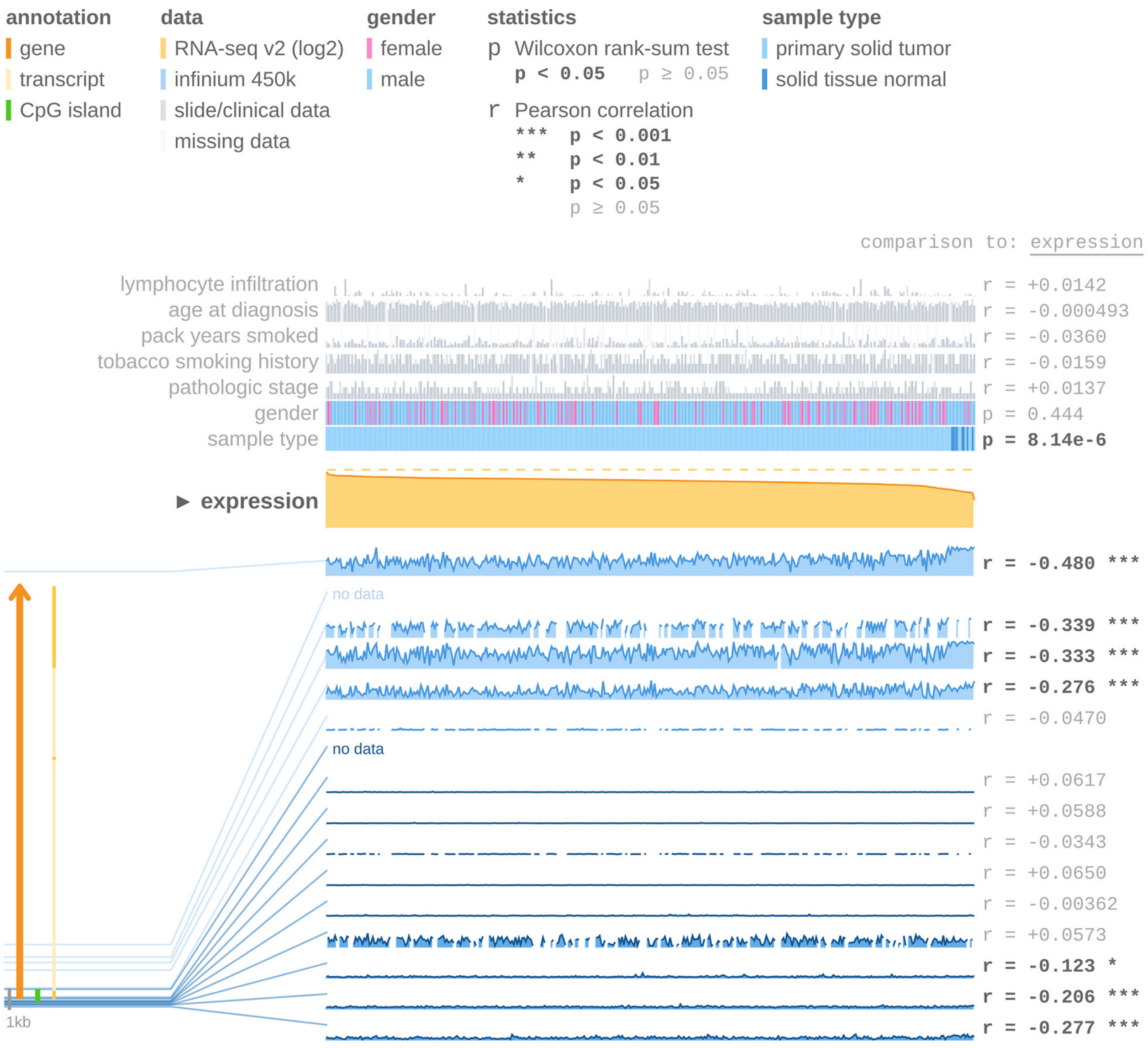

Supplementary Figure 10: Methylation analysis of *Perp* gene in Lung squamous cell carcinoma. The highlighted probes are promoter gene region.

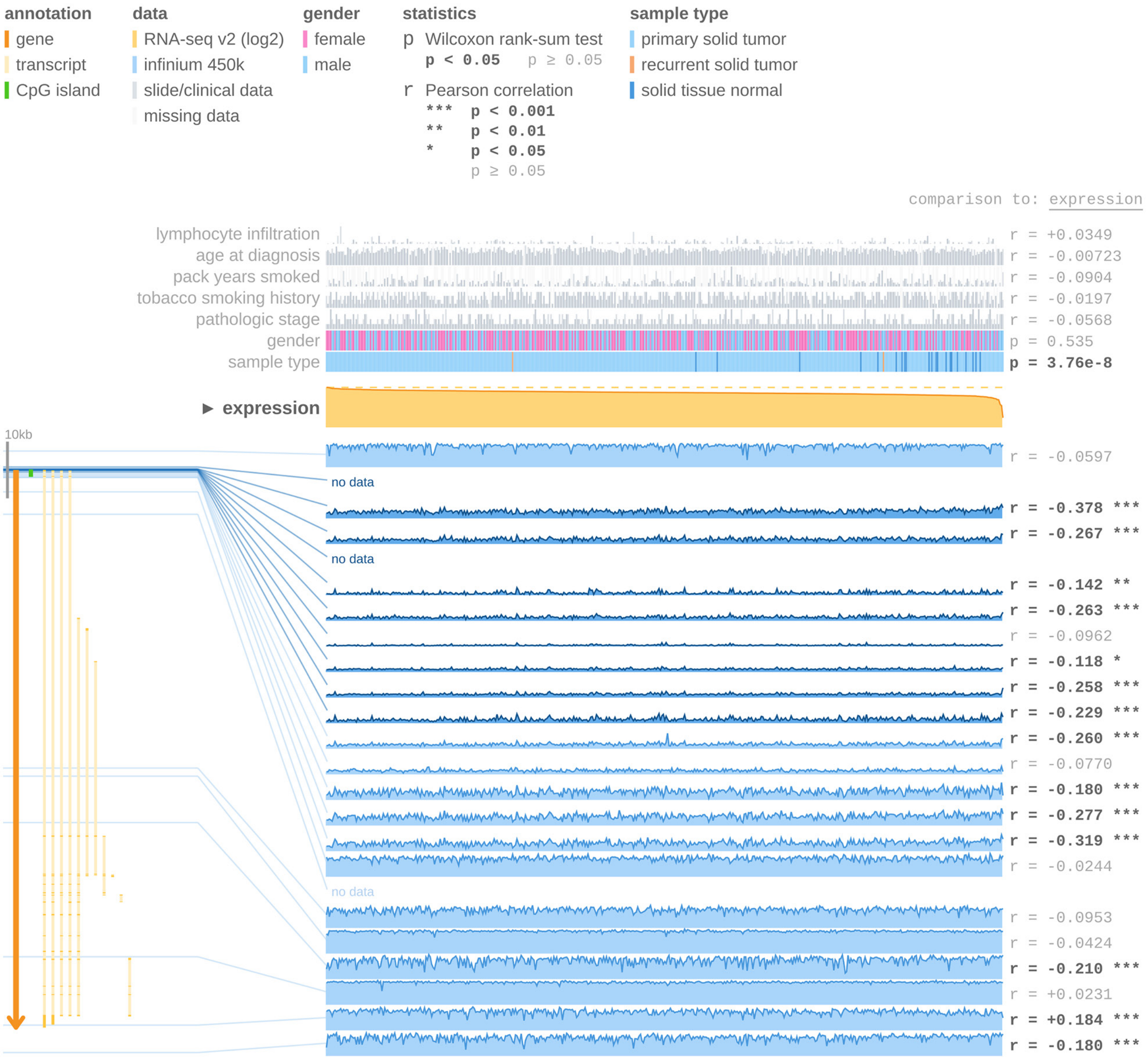

Supplementary Figure 11: Methylation analysis of *Cdh1* gene in lung adenocarcinoma. The highlighted probes are promoter gene region.

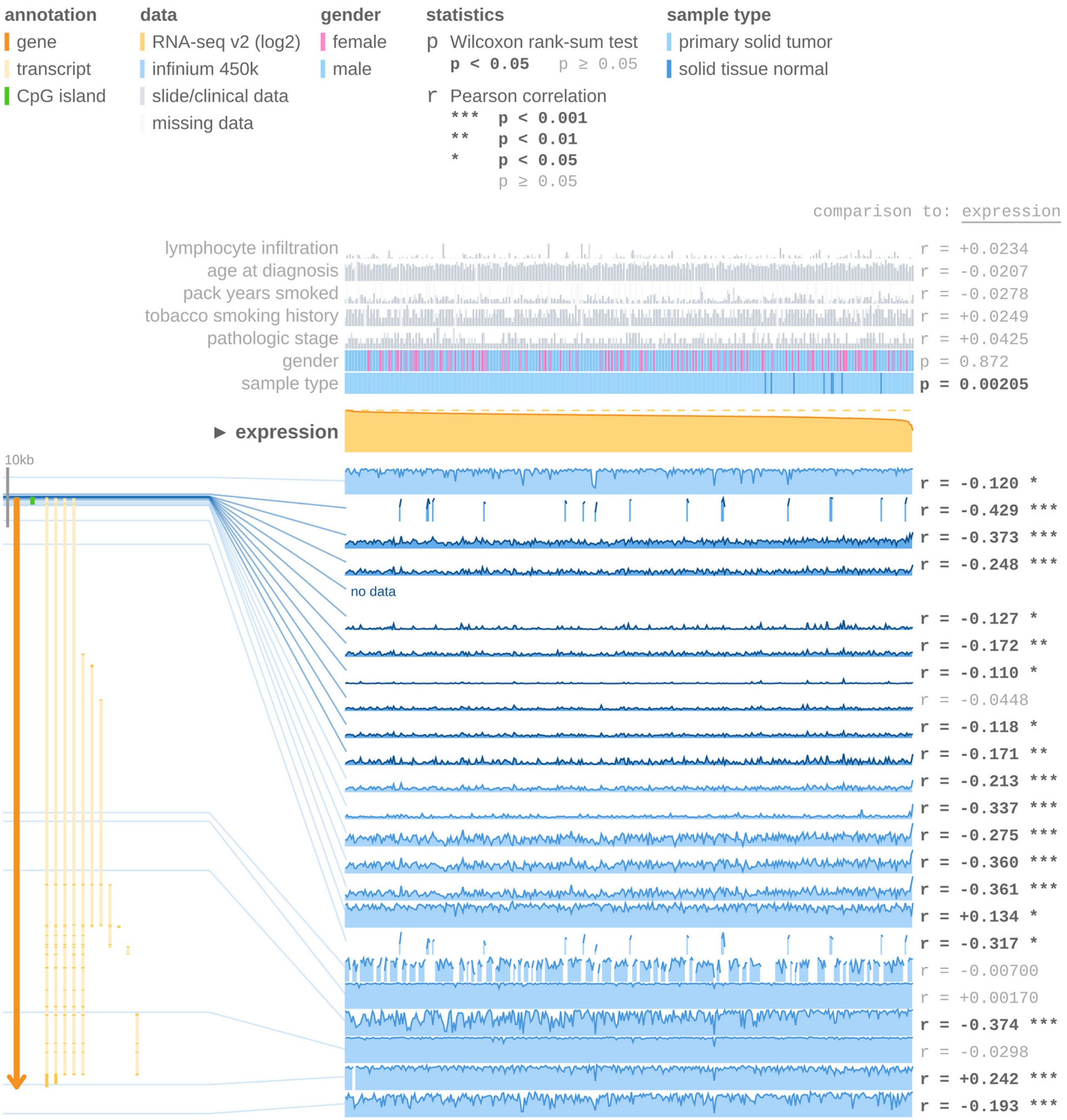

Supplementary Figure 12: Methylation analysis of *Cdh1* gene in Lung squamous cell carcinoma. The highlighted probes are promoter gene region.

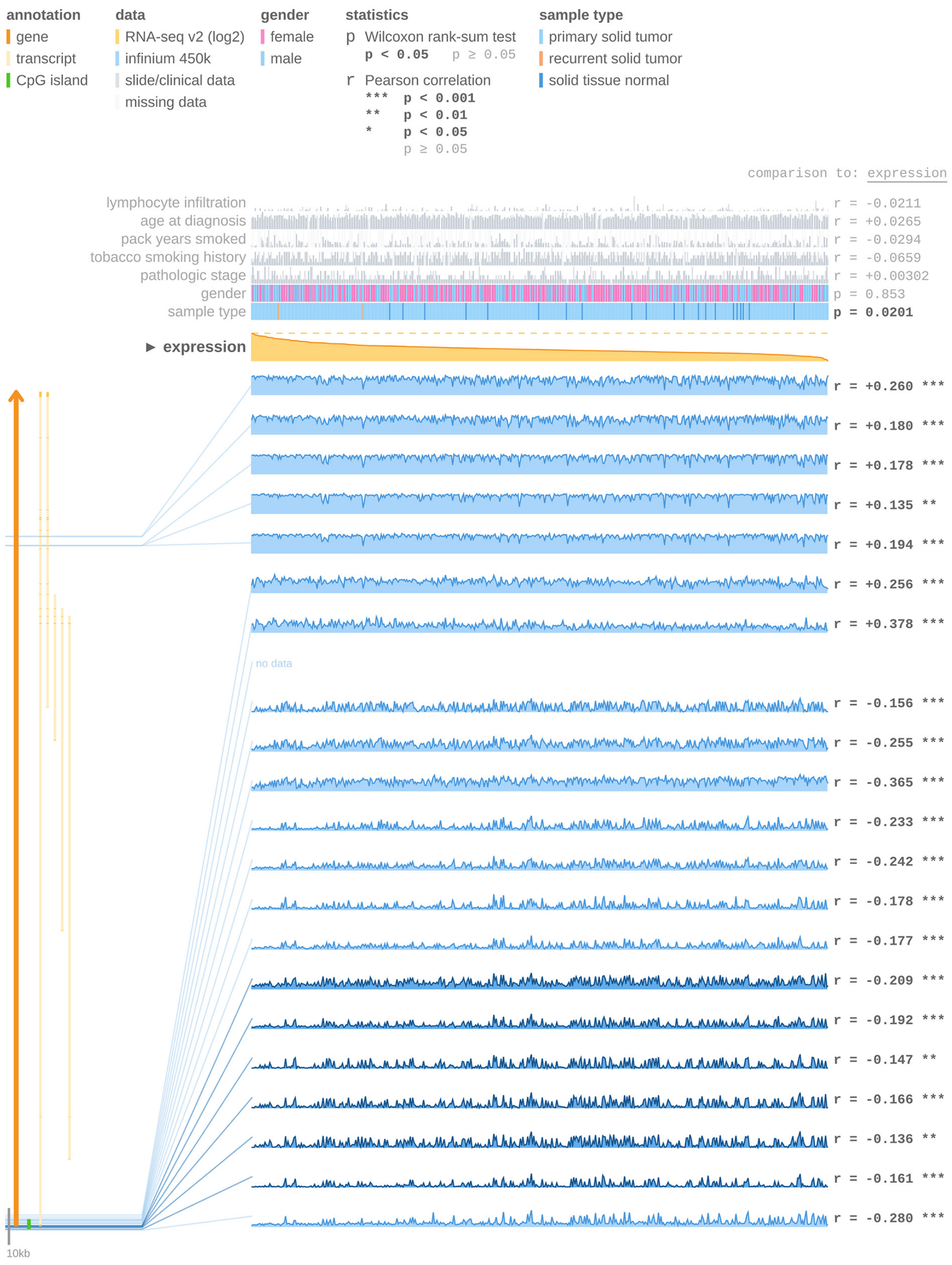

Supplementary Figure 13: Methylation analysis of *Cdh2*gene in lung adenocarcinoma. The highlighted probes are promoter gene region.

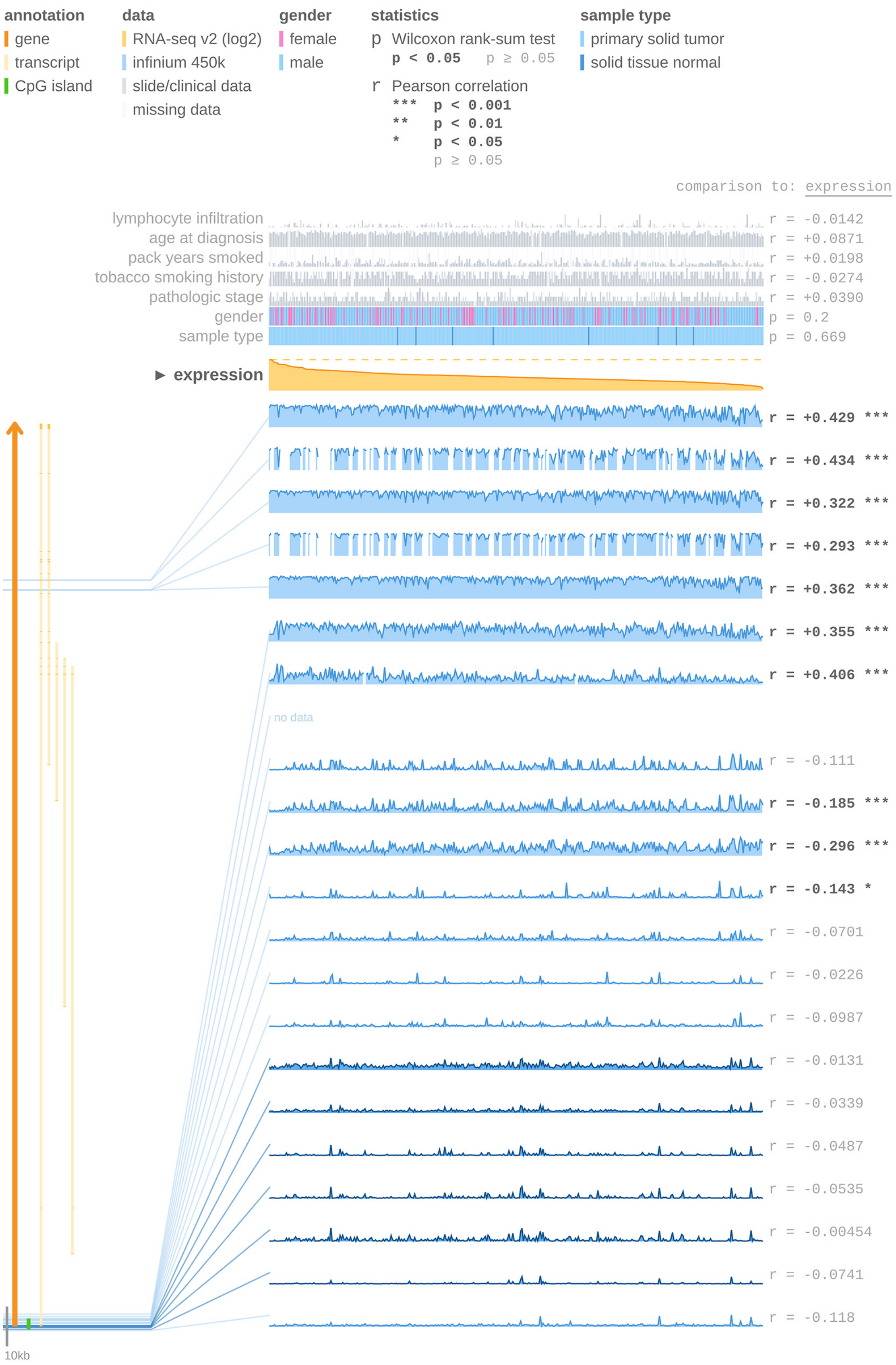

Supplementary Figure 14: Methylation analysis of *Cdh2* gene in Lung squamous cell carcinoma. The highlighted probes are promoter gene region.

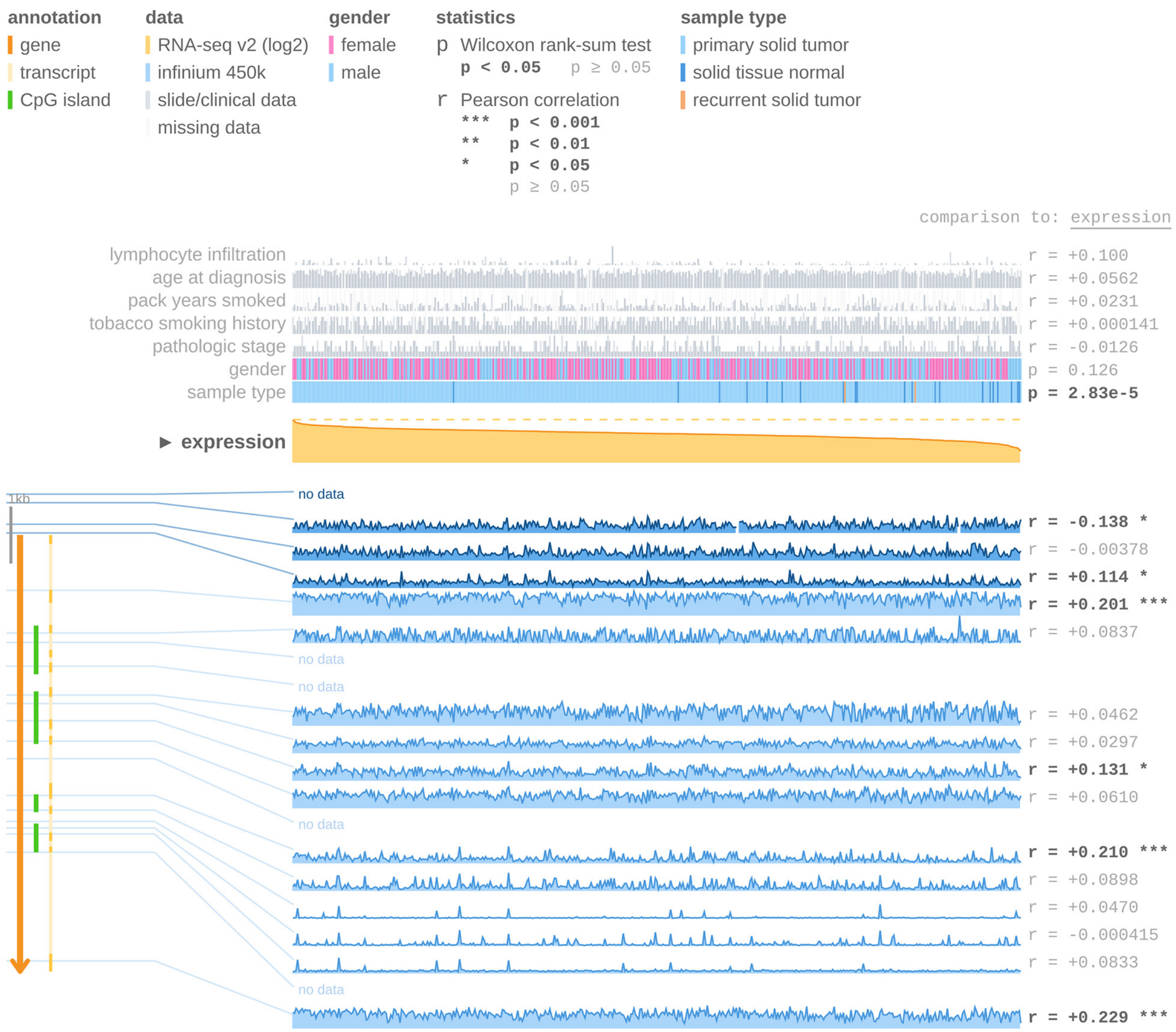

Supplementary Figure 15: Methylation analysis of *Mmp9* gene in lung adenocarcinoma. The highlighted probes are promoter gene region.

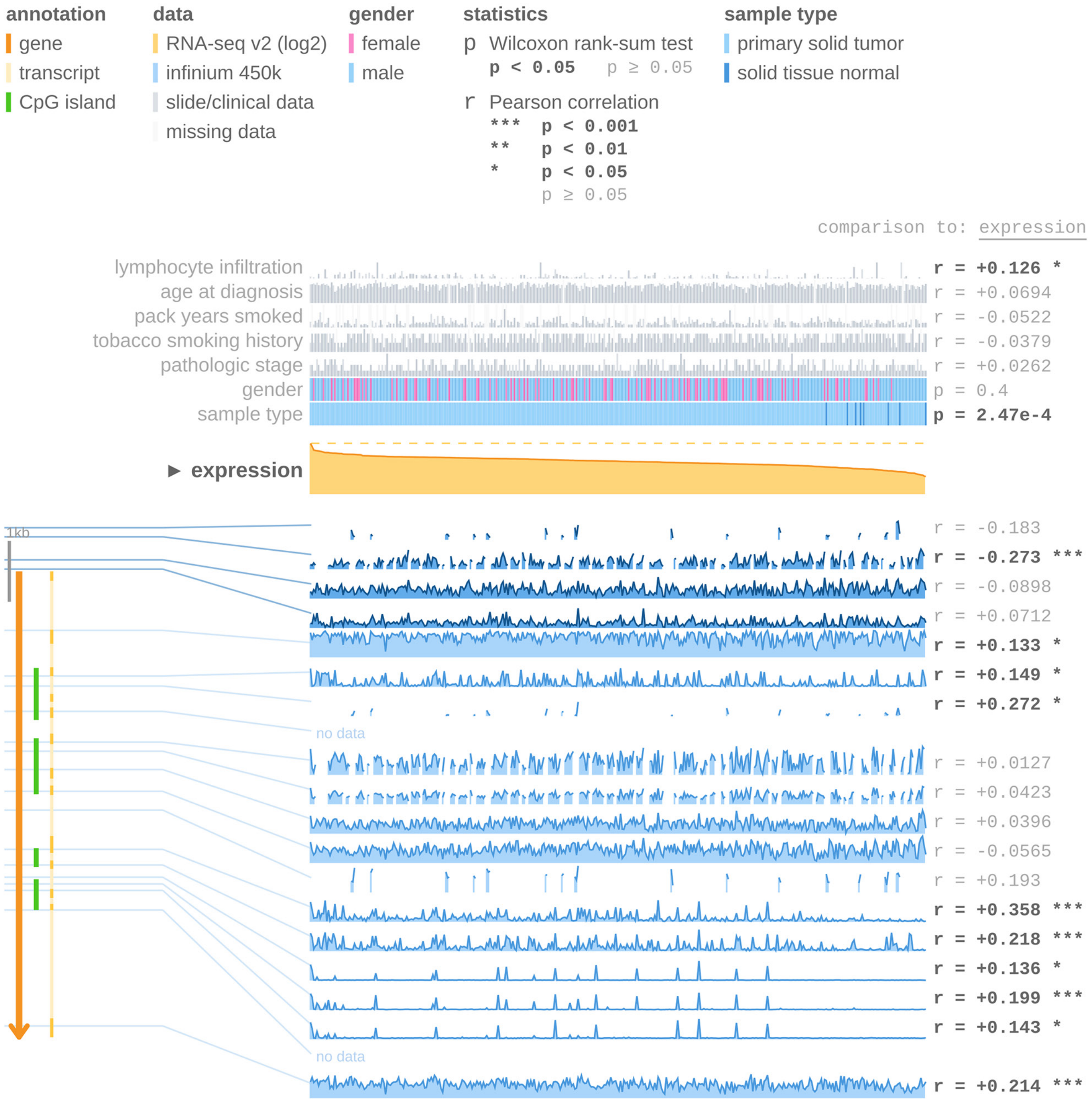

Supplementary Figure 16: Methylation analysis of *Mmp9* gene in Lung squamous cell carcinoma. The highlighted probes are promoter gene region.

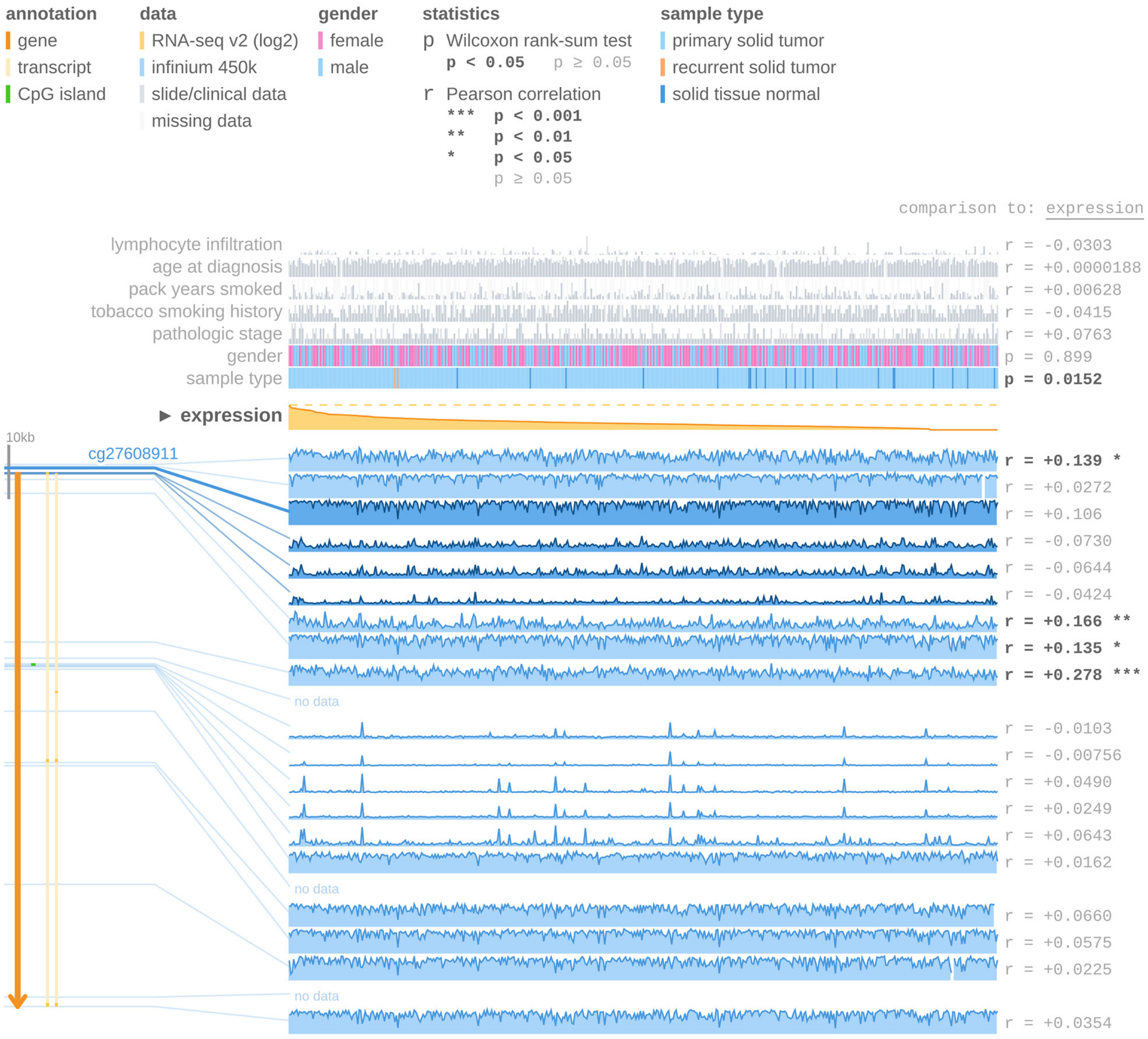

Supplementary Figure 17: Methylation analysis of *Gap43* gene in lung adenocarcinoma. The highlighted probes are promoter gene region.

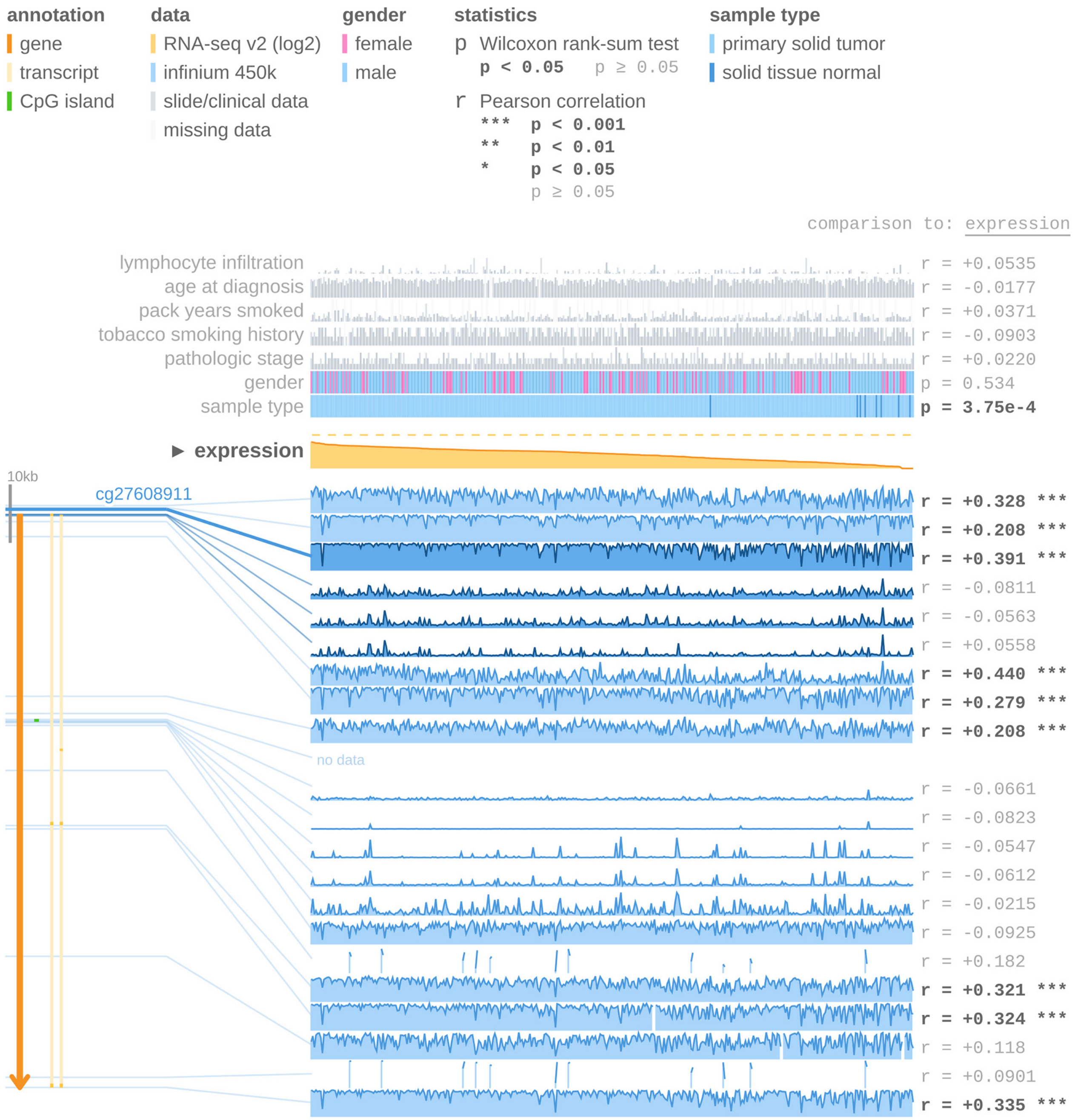

Supplementary Figure 18: Methylation analysis of *Gap43* gene in Lung squamous cell carcinoma. The highlighted probes are promoter gene region.

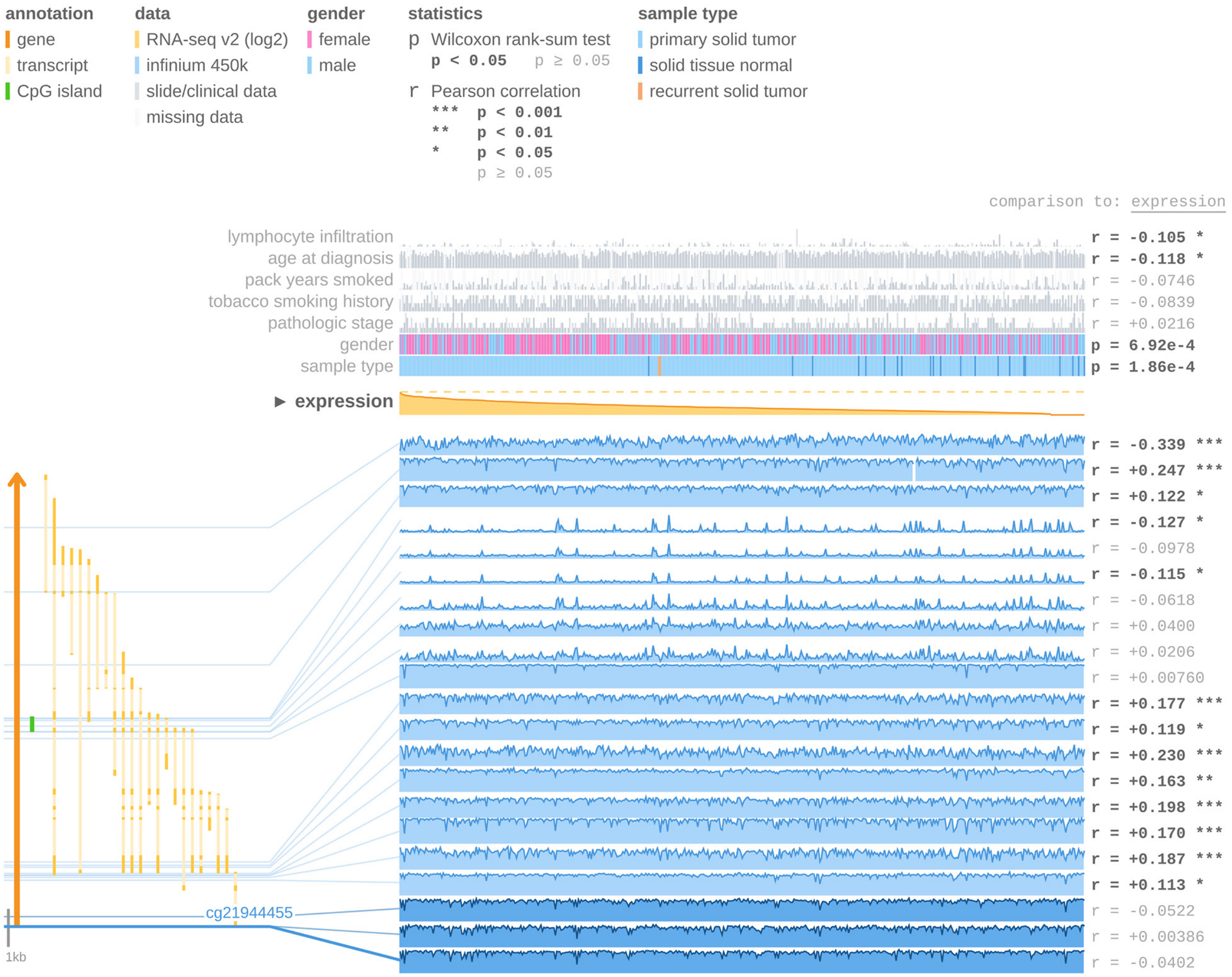

Supplementary Figure 19: Methylation analysis of *Gfap* gene in lung adenocarcinoma. The highlighted probes are promoter gene region.

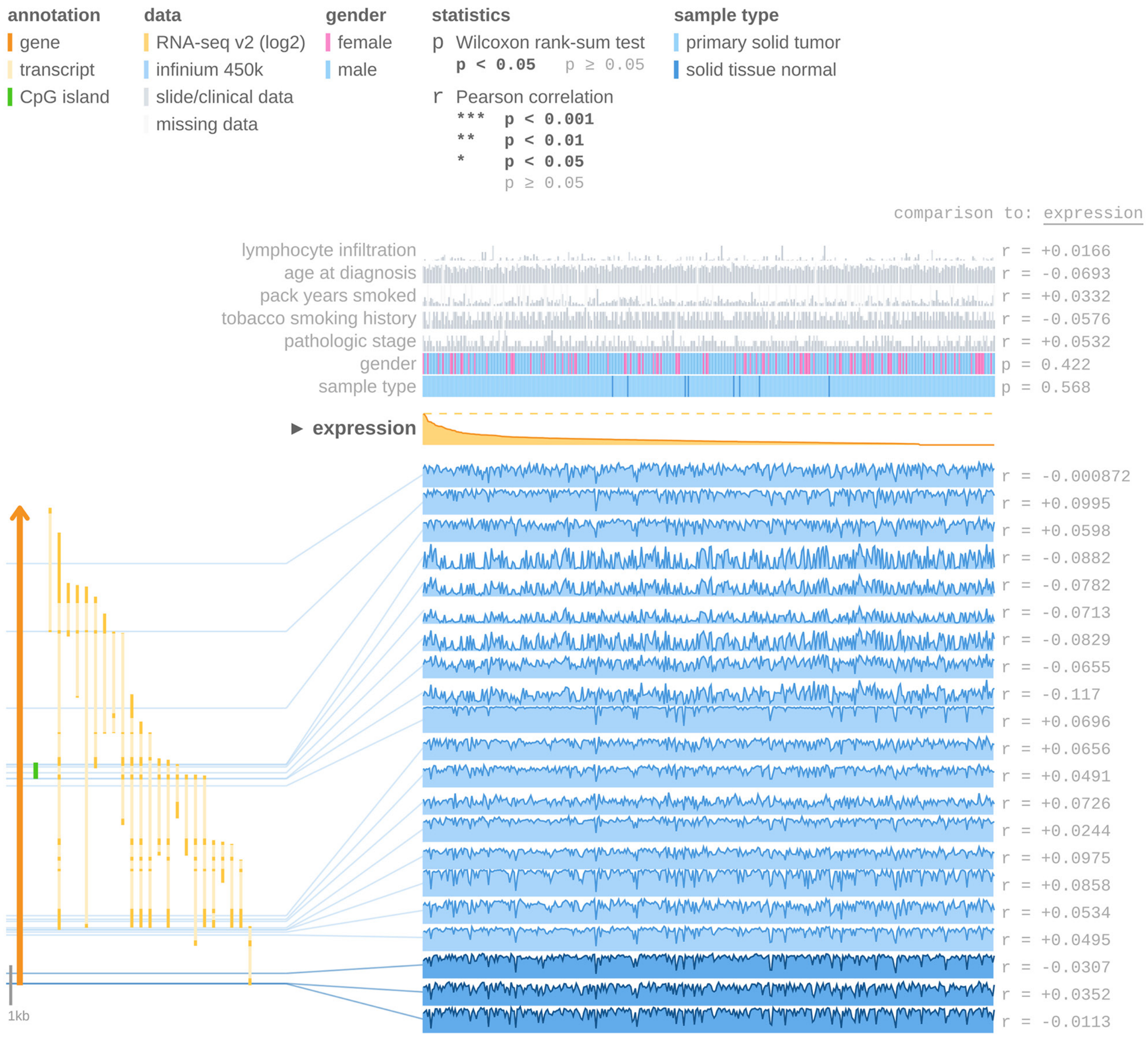

Supplementary Figure 20: Methylation analysis of *Gfap* gene in Lung squamous cell carcinoma. The highlighted probes are promoter gene region.

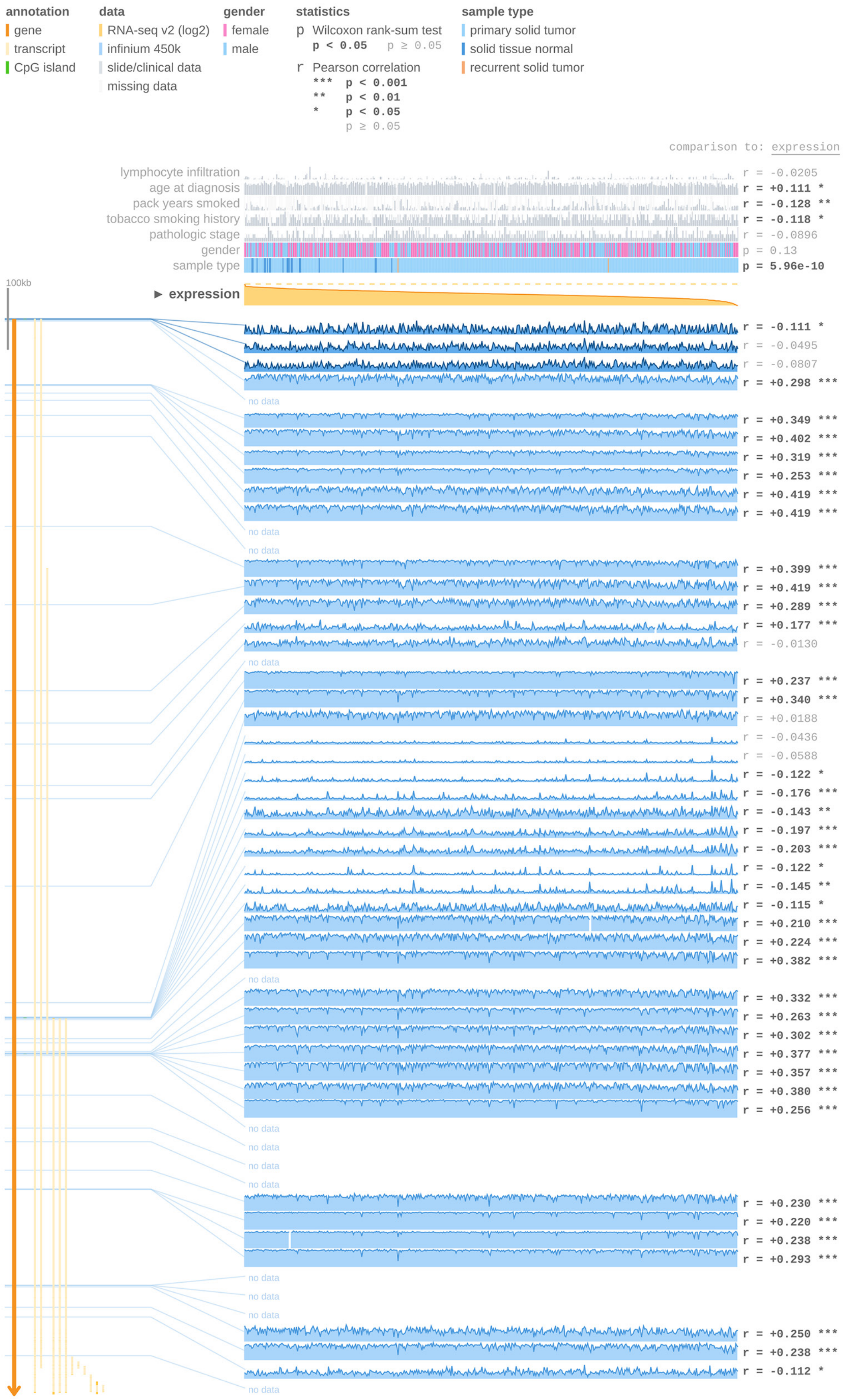

Supplementary Figure 21: Methylation analysis of *Robo2* gene in lung adenocarcinoma. The highlighted probes are promoter gene region.

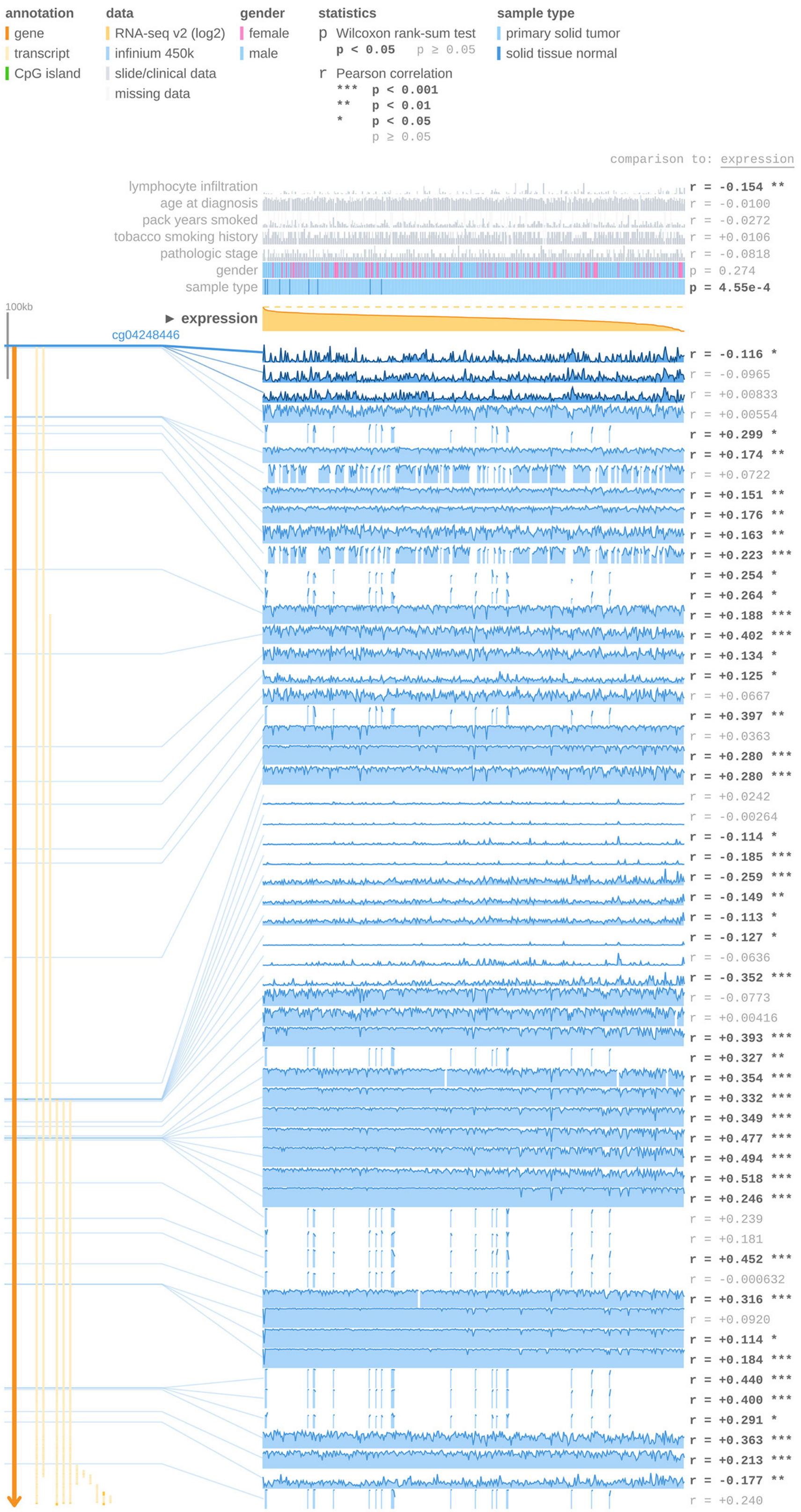

Supplementary Figure 22: Methylation analysis of *Robo2* gene in Lung squamous cell carcinoma. The highlighted probes are promoter gene region.

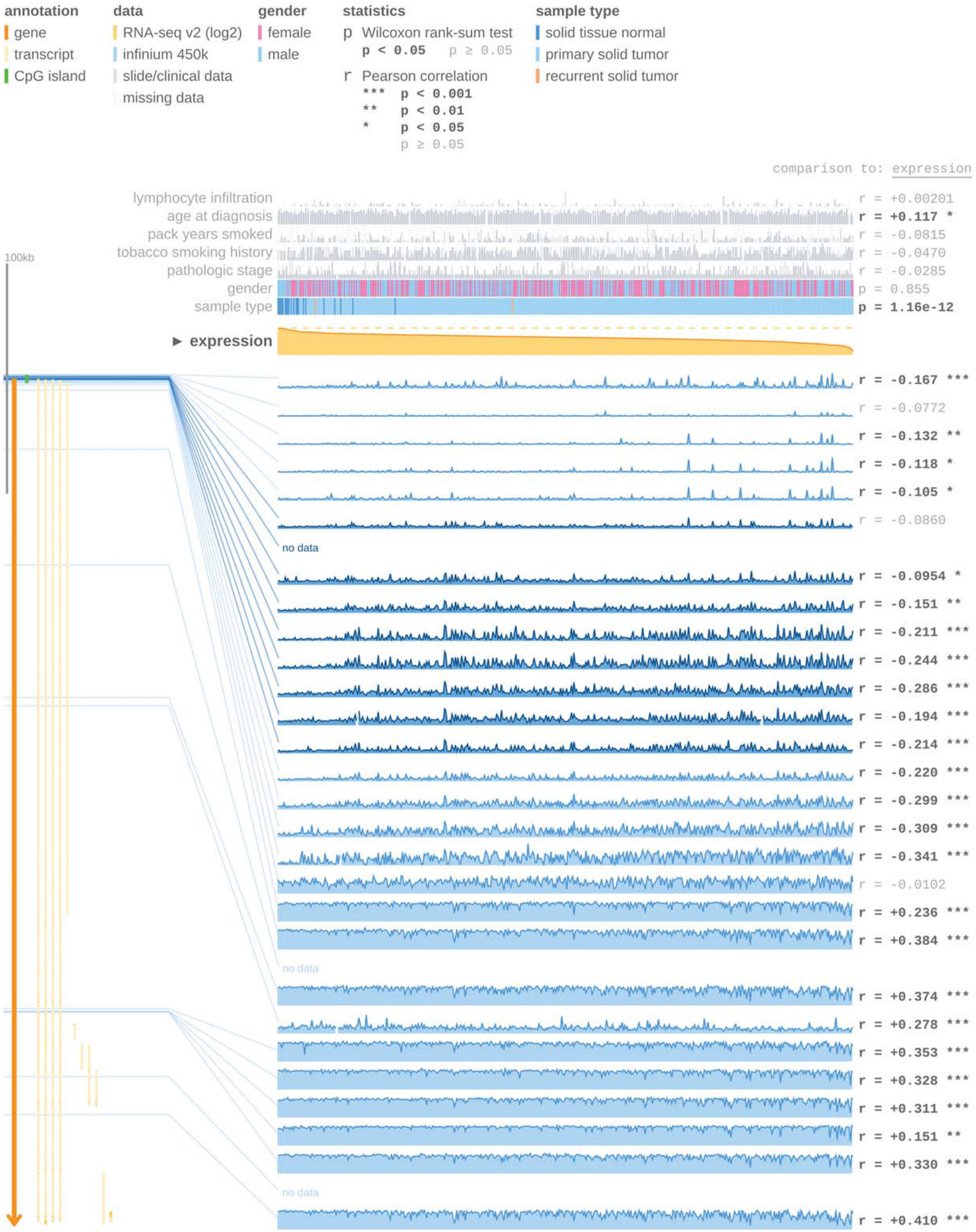

Supplementary Figure 23: Methylation analysis of *Slit2* gene in lung adenocarcinoma. The highlighted probes are promoter gene region.

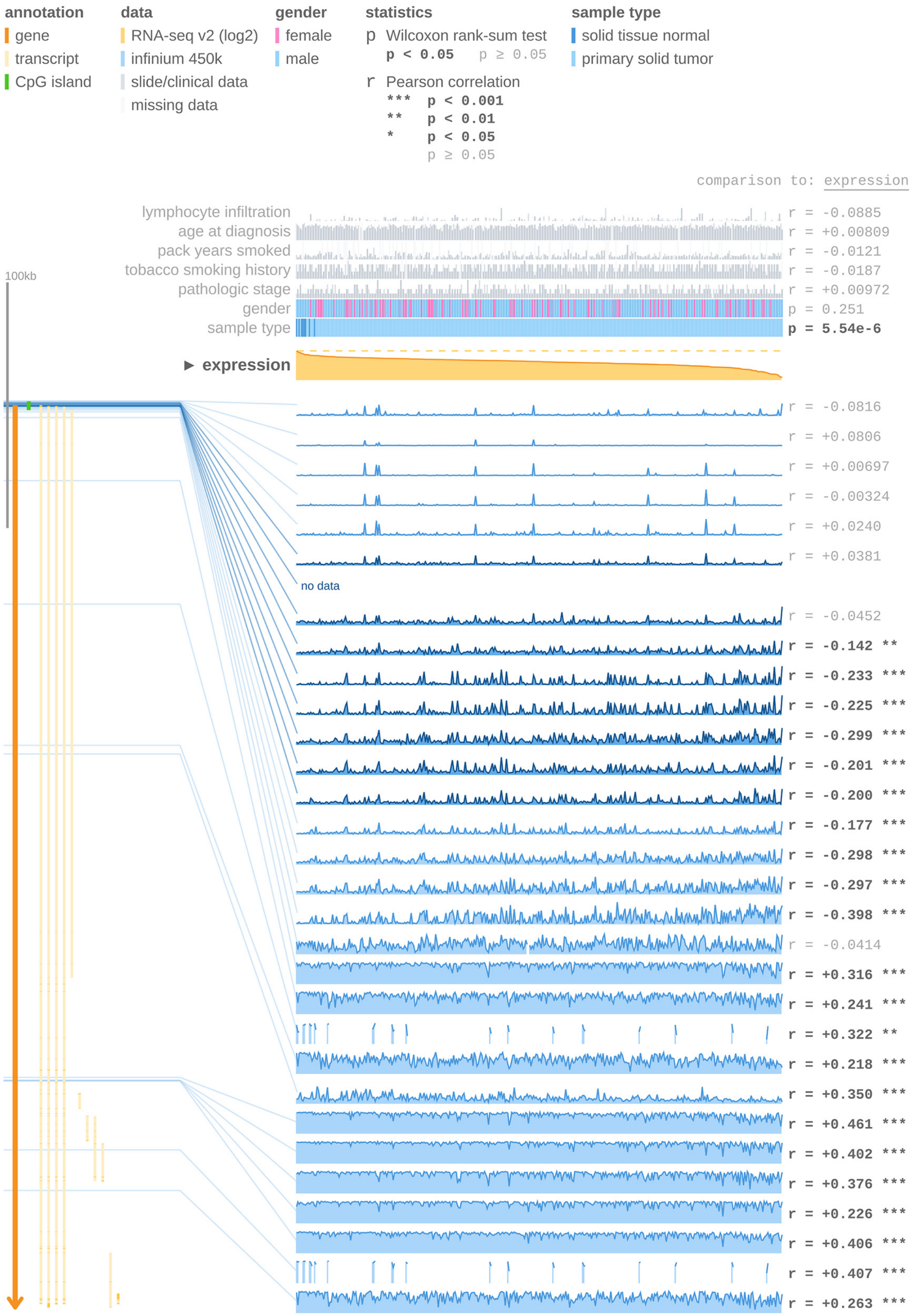

Supplementary Figure 24: Methylation analysis of *Slit2* gene in Lung squamous cell carcinoma. The highlighted probes are promoter gene region.

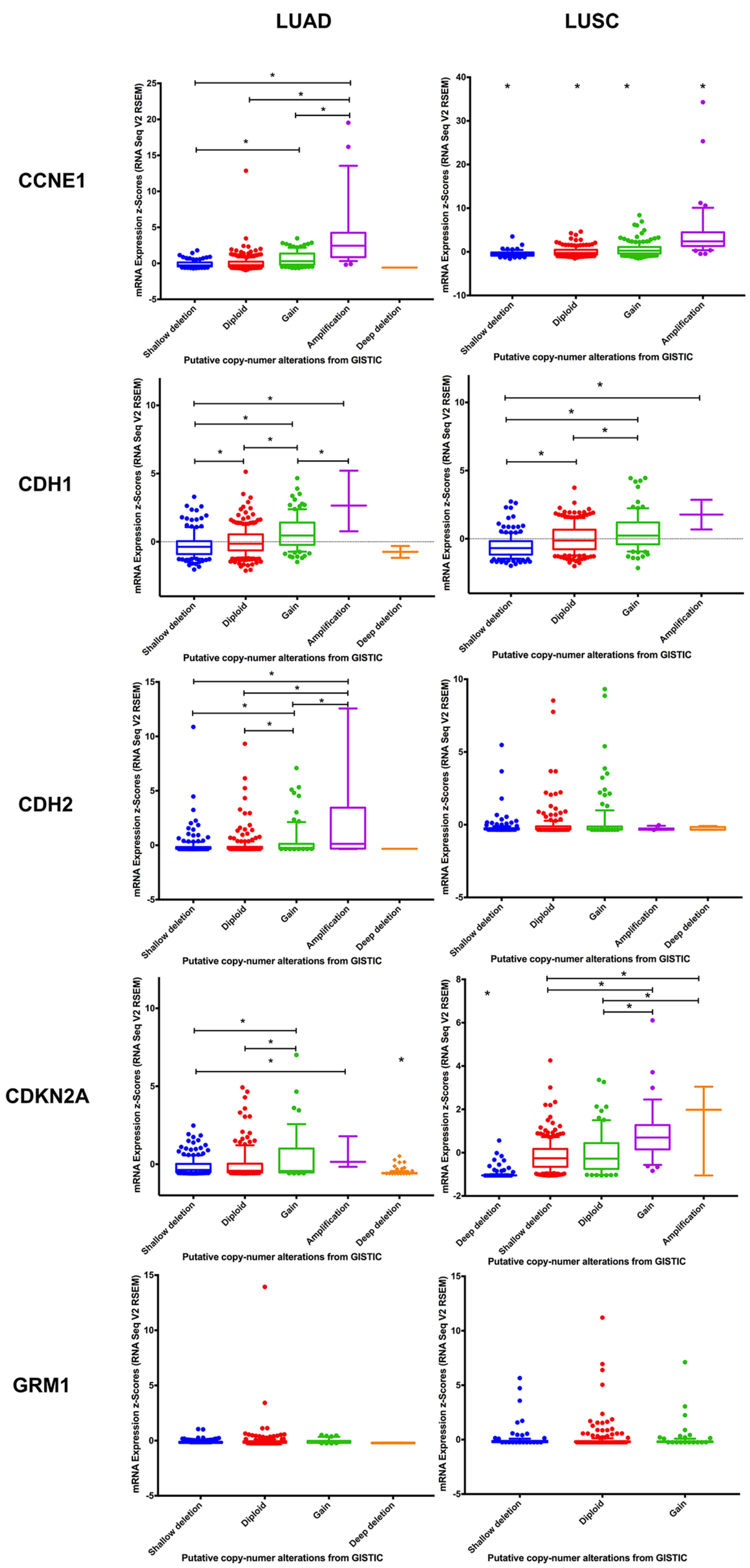

Supplementary Figure 25: Copy number alteration and mRNA expression analysis in lung adenocarcinoma and lung squamous cell carcinoma.

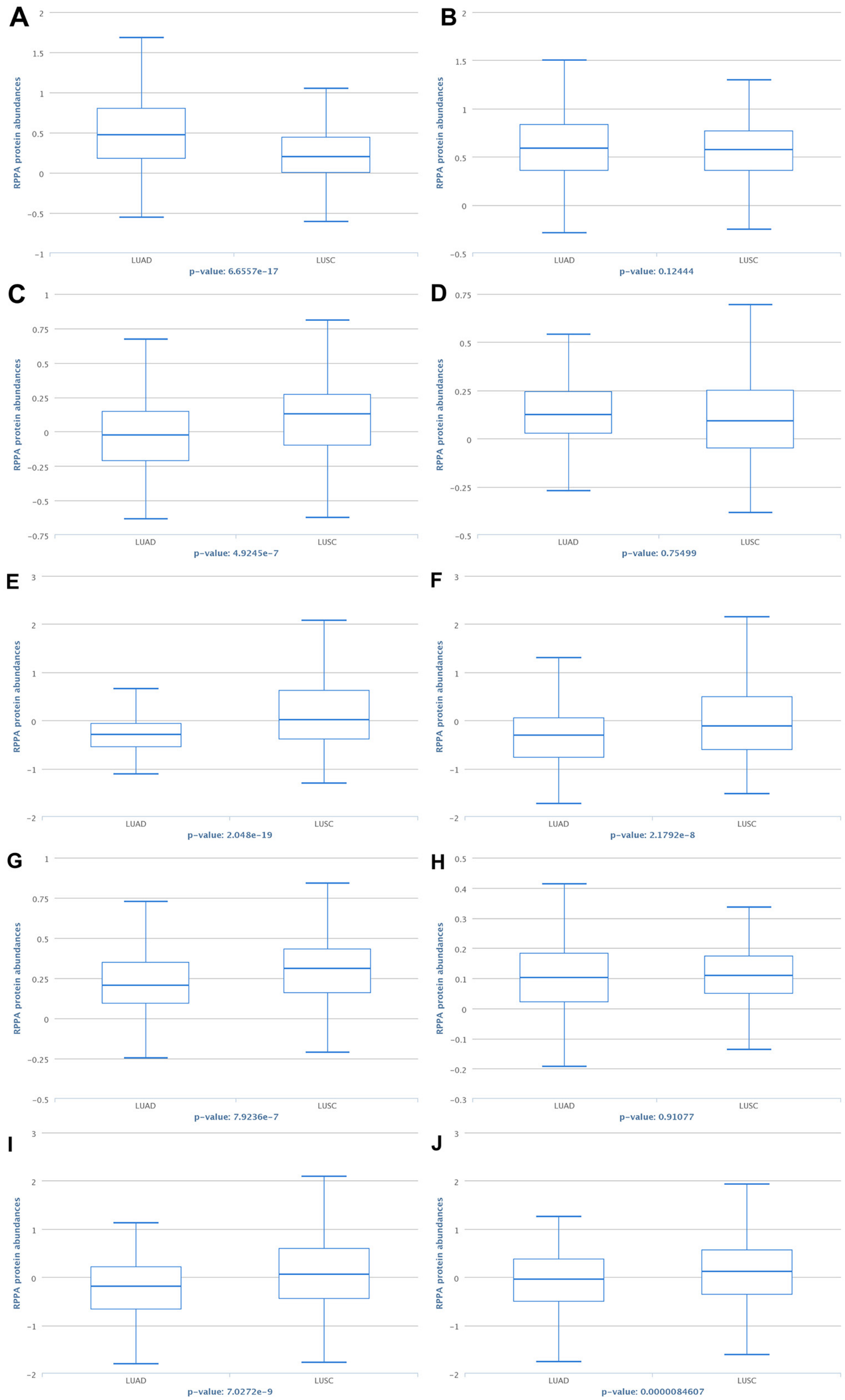

Supplementary Figure 26: RRPA analysis of MEK1 **(A)**, MEK1_pS217S221 **(B)**, Erk2 **(C)**, PRAS40_pT246 **(D)**, AKT_pT308 **(E)**, AKT_pS473 **(F)**, CRAF **(G)**, CRAF_pS338 **(H)**, GSK3_pS9 **(I)**, GSK3ALPHABETA_pS21S9 **(J)** proteins in lung adenocarcinoma and lung squamous cell carcinoma.

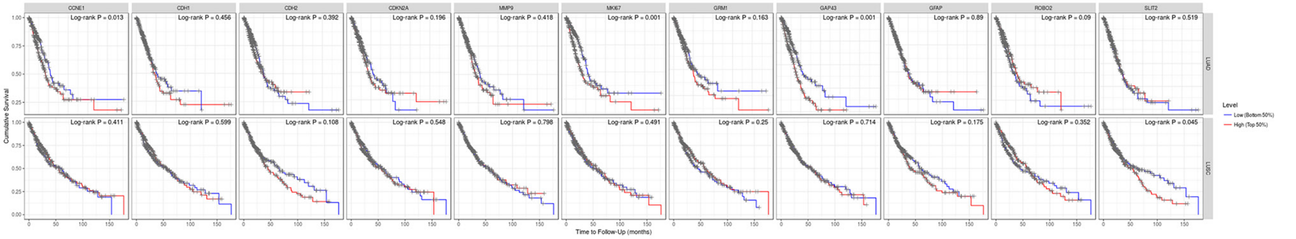

Supplementary Figure 27: TIMER Survival analyses in lung adenocarcinoma (LUAD) and lung squamous cell carcinoma (LUSC). Upregulation of *Ccne1*, *Mki67* and, *Gap43* downregulation of *Robo2* are associated with poor survival in LUAD. Upregulation of *Slit2* is associated with poor survival in LUSC.
